# New biologically relevant resistance to bacterial wilt in heat-stressed tomato revealed by two-reference Genome Wide Association

**DOI:** 10.1371/journal.pgen.1011820

**Published:** 2026-08-13

**Authors:** Adrien Belny, Henri Desaint, Mylène Rigal, Sébastien Carrère, Jérôme Gouzy, Gregori Bonnet, Céline Labourey, Elise Albert, Laurent Grivet, Fabrice Roux, Fabienne Vailleau, Tristan Mary-Huard, Richard Berthomé

**Affiliations:** 1 Univ Toulouse, CNRS, INRAE, LIPME, Castanet-Tolosan, France; 2 SYNGENTA Seeds, Saint-Sauveur, France; 3 SYNGENTA France SAS, Sarrians, France; 4 Université Paris-Saclay, INRAE, CNRS, AgroParisTech, GQE - Le Moulon, Gif-sur-Yvette, France; 5 Université Paris-Saclay, AgroParisTech, INRAE, UMR MIA Paris-Saclay, Palaiseau, France; Université Paris-Saclay: Universite Paris-Saclay, FRANCE

## Abstract

Ongoing climate change is driving unprecedented environmental fluctuations, with extreme events predicted to increase in frequency, intensity and duration. Among climatic parameters, temperature is expected to fluctuate the most by the end of the century and its elevation has already demonstrated to pose a major challenge to plant health. In this context, understanding how temperature modulates plant-pathogen interactions and their underlying genetic architecture is critical. Bacterial wilt, caused by strains of the *Ralstonia solanacearum* species complex, is a devastating disease affecting many plant species. Genetic resistance remains the most effective control strategy. In tomato, resistance is quantitative and mostly relies on quantitative trait loci (QTLs) *bwr-6* and *bwr-12*. However, as in other crops, high temperature and humidity can compromise this resistance in commercial tomato cultivars. We investigated temperature-dependent quantitative disease resistance (QDR) using a panel of 189 wild tomato accessions, predominantly *Solanum pimpinellifolium*, representing genetic diversity from contrasting ecological conditions. Disease progression was monitored from three to ten days post-inoculation at 28 °C and 32 °C, using a time-course phenotyping approach. Genome-wide association (GWA) analyses were performed, based on daily symptom scores and two reference genomes, to account for structural variations and improve QTL detection. This strategy identified 44 candidate genes and revealed a temporally dynamic genetic architecture of the plant response. Strikingly, no candidate genes were shared between temperatures, supporting distinct genetic determinants under different temperature conditions. Many candidate genes were expressed in roots and belong to gene families involved in plant immunity, with two candidates co-localizing with *bwr-6* and *bwr-12*, whose causal genes remain unknown. Altogether, our findings demonstrate that resistance to bacterial wilt in wild tomato is flexible, environment-dependent, and temporally dynamic, highlighting the importance of integrating environmental context and genomic diversity to better understand plant-pathogen interactions.

## Author summary

Climate change increases temperatures and creates more extreme environmental conditions, which can worsen plant diseases and threaten crop production. It has been reported that major plant diseases become more severe at higher temperatures. However, little is known about how plants maintain resistance under such conditions. In this study, we investigated how wild tomato plants respond to bacterial wilt at two different temperatures. We assembled a panel of 189 accessions, mostly *Solanum pimpinellifolium*, representing genetic diversity from contrasting habitats. The panel was inoculated and disease progression was monitored over time to capture both early and late responses to *Ralstonia pseudosolanacearum* at 28 °C and 32 °C. By analyzing genetic variation across these accessions, we identified 44 candidate genes associated with resistance and discovered that different sets of genes are mobilized depending on the temperature. Many of these genes are linked to immunity and are expressed in roots, where infection occurs. These results demonstrate that resistance is flexible and depends on environmental conditions. They also suggest that previously pyramided resistance genes may become less effective in the short or medium term, highlighting the importance of studies like this to accurately identify the genes and mechanisms that should be targeted for future breeding efforts.

## Introduction

A growing number of studies have assessed various combinations of plant-pathogen interactions under abiotic stress conditions (cold, high temperature, low nitrogen, drought) [1,2], showing that many plant defense responses are negatively affected by such constraints. However, the underlying mechanisms remain largely unknown, and most studies are descriptive and primarily rely on phenotypic and transcriptomic analyses [1,3]. Among climatic parameters, temperature is predicted to fluctuate the most by the end of the century [4], and a meta-analysis showed that 55% of 145 genetic resistance sources are compromised under heat stress, leading to increased disease severity [3]. In this context, understanding how climatic parameters affect host and pathogen responses as well as identifying novel robust resistance or mechanisms that ensure plant resilience under fluctuating environmental conditions is crucial.

Bacteria belonging to the *Ralstonia solanacearum* species complex (RSSC) are able to infect a wide range of plant species from 53 botanical families and are responsible for various bacterial diseases that can lead to considerable economic losses in major crops (tomato, potato, eggplant and other Solanaceae species), crops of local importance (banana, peanuts, etc), and ornamentals (pelargonium, rose) [5,6]. Strains of the RSSC are soil-borne pathogens that enter plant roots, colonize and multiply in the xylem, clogging vessels and leading to characteristic wilting symptoms [7]. These strains originate from tropical and sub-tropical regions and are organized into four major genetic clusters, called phylotypes. Interestingly, the geographical origins of the strains are correlated with their phylotype [5]. To limit the occurrence of bacterial wilt and overcome disease outbreaks, integrated pest management combines multiple strategies [8]. Promising developments in control methods are based on, or use, living organisms [9,10] and can complement traditional cultivation practices, such as crop rotation [11] and pesticide use [8]. However, multiple studies have shown that genetic solutions remain the most efficient, cost-effective and eco-friendly means of tackling these diseases [12,13].

To date, more than forty QTLs underlying plant defense responses to different strains of RSSC have been mapped in *A. thaliana*, tobacco, potato, peanut, pepper, eggplant and tomato, mainly through traditional QTL mapping approaches [13,14]. Analyses of host resistance together with studies of the RSSC type III effectors (T3E) repertoire, a major determinant of pathogenicity, indicate that plant immunity to RSSC is generally polygenic and strain-specific [12,13]. Despite the identification of numerous resistance loci, their underlying molecular mechanisms remain poorly understood. One exception is the *A. thaliana* nucleotide-binding, leucine-rich repeat (NLR) receptor pair RPS4/RRS1 [15–17], which confers qualitative resistance to several RSSC strains [15,18]*,* as well as to other pathogens [19] and contributes to QDR against *Xanthomonas campestris* pv*. campestris* strain CFBP6943 and GMI1000 at 27 °C [20,21]. Importantly, both RPS4/RRS1-mediated resistance and broader defense responses are compromised in case of GMI1000 at 30 °C, highlighting the strong impact of elevated temperature on plant immunity to this pathogen [21]. Natural variation in plant-pathogen interactions coupled with GWAS have proven effective in detecting QTL underlying disease resistance [22–25]. In Arabidopsis, GWAS analyses of responses to GMI1000 and four related T3E mutant strains at 27°C detected several susceptibility genes, some of which were functionally validated [21,26,27]. Nevertheless, such strategies investigating plant responses under combined biotic and abiotic stress conditions remain scarce. Using 176 *A. thaliana* accessions inoculated with GMI1000 under controlled conditions at 27 °C and 30 °C, with different root-wounding regimes, recent GWAS revealed distinct resistance mechanisms and temporal plasticity in plant responses. Functional validations further identified *Strictosidine Synthase-Like Proteins* (*SSL4* and *SSL5*), *Solo Dancers* (*SDS*), and *Cellulose Synthase A3* (*CESA3*, subunit 3 of the primary cell wall cellulose complex) genes as susceptibility factors contributing to bacterial wilt development under elevated temperature conditions [21,28,29].

Tomato (*Solanum lycopersicum*) is one of the main Solanaceae species cultivated worldwide and serves as a model for fruit-bearing crops. As natural hosts of *R. solanacearum*, most tomato cultivars have been described to be susceptible to a wide range of strains belonging to the four phylotypes of the RSSC [30]. *Solanum pimpinellifolium*, a wild relative of cultivated tomato, has contributed to the identification of resistance to over 45 different diseases [31,32] including resistance to *Phytophthora infestans* [33,34], *Fusarium oxysporum* (*I2* gene) [35], gray leaf spot disease (*Sm* gene) [36] and bacterial speck (*Pto* gene) [37]. For bacterial wilt, the *S. lycopersicum* ‘Hawaii 7996’ cultivar is still the most tolerant to several strains of *R. solanacearum* [38] and is widely used in breeding programs. Its tolerance originates from *S. pimpinellifolium* accession PI127805A and relies on at least seven QTLs, of which *bwr-6* and *bwr-12* are the most studied [39]. However, none of the genes underlying these QTLs have been cloned and the genetic architecture of this QDR appears complex: *bwr-6* and *bwr-12* each comprise multiple smaller QTLs, four for *bwr-6* and three for *bwr-12* [40]. As for other Solanaceae species defense responses, the “Hawaii 7996” cultivar tolerance appears sensitive to elevated temperatures [41,42]. Increased soil temperatures can impair the bacterial wilt QDR for some tomato varieties [43]. In Taiwan and other parts of South-East Asia, varieties with *bwr-12*-based QDR tend to be more susceptible to the bacteria at high temperatures. When phenotyped under heat stress, the ‘Hawaii 7996’ cultivar exhibited a wilting rate ranging from 28% to 52%, depending on the trial [38]. These observations demonstrate the need to identify and characterize new sources of resistance to bacterial wilt that remain effective under elevated temperature or changing environmental conditions.

Tomato wild relatives originated from tropical and equatorial regions and evolved under diverse and often extreme environmental conditions, probably in contact with *R. solanacearum* species [2]*.* While optimal growth conditions for tomato vary between 13 °C and 33 °C depending on the variety studied [43], the optimal growth temperature for the *R. pseudosolanacearum* GMI1000 strain is 28 °C [44]. As tomato domestication led to a genetic bottleneck and reduced intraspecific diversity [45], exploring closely related wild species from contrasting habitats offers the opportunity to uncover adaptive traits that may have been lost during domestication. We therefore hypothesized that accessions naturally exposed to the bacterium could have evolved defense mechanisms shaped by their native environmental conditions. To investigate how temperature modulates QDR to *R. pseudosolanacearum* and identify its genetic bases, we implemented a combined phenotyping and genomics approach using contrasting temperature conditions. A panel of wild tomato accessions, enriched in *S. pimpinellifolium*, was challenged with the GMI1000 strain under permissive and elevated temperatures. Disease progression was monitored through a time-course experiment, enabling the capture of both early and late responses to infection. All accessions were sequenced and analysed using a GWAS approach based on two distinct reference genomes. These references were selected to reflect the genetic diversity of a previously described panel [46], for their contrasting biomes of origin and responses to the bacteria at 28 °C. This strategy allowed us to dissect the contribution of genetic variation, including structural differences between genomes, to the architecture of resistance across environments. Our study highlights the temporal dynamic and different genetic architectures of the plant defense response at 28 °C and 32 °C, with a strong impact of the temperature elevation. Our findings also contribute to refining the detection of two previously detected major QTLs: *bwr-6* and *bwr-12*, for which genes have not been identified.

## Results

### Panel characteristics

**Fig 1:**
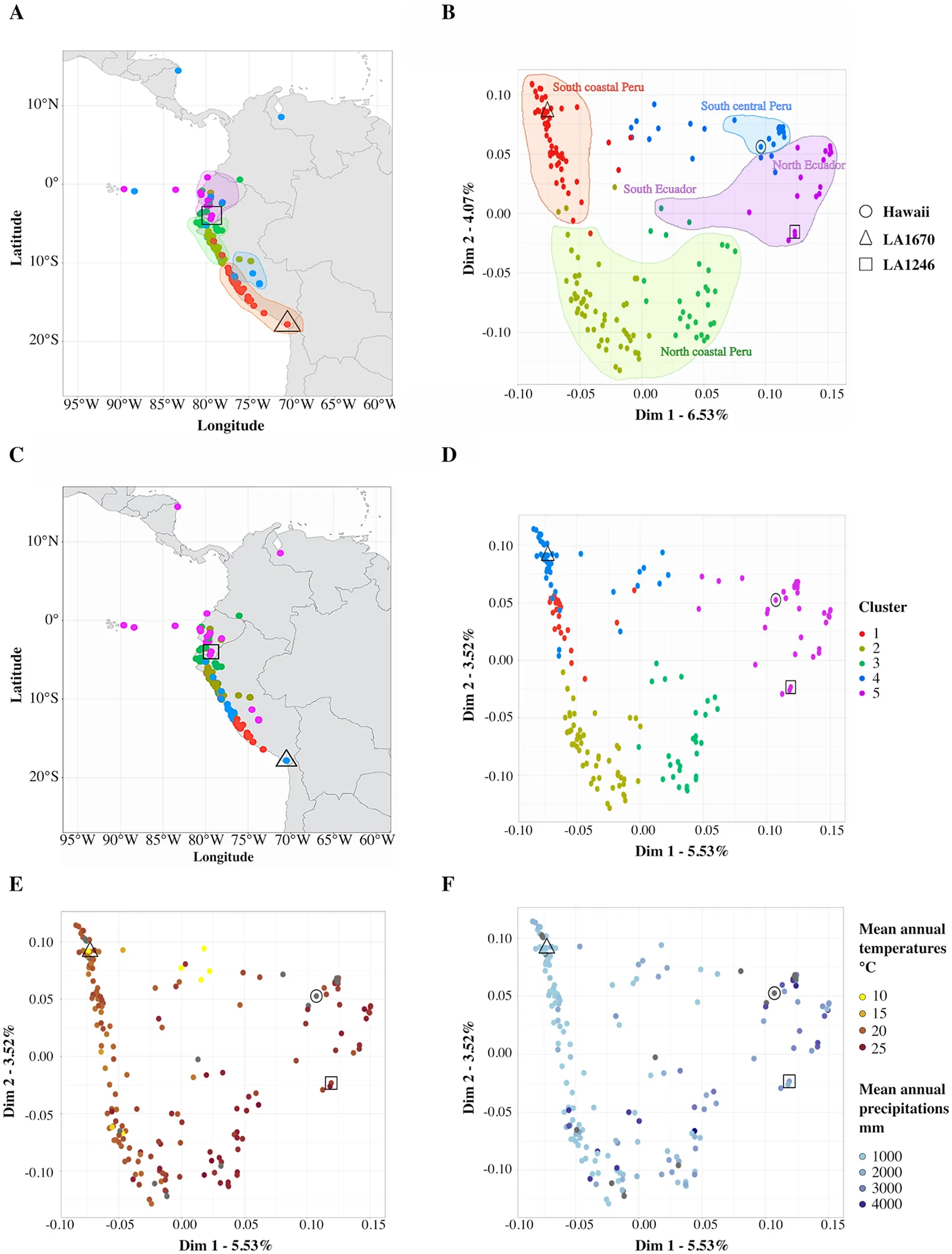
Geographic distribution and population structure of accessions based on LA1670 and LA1246 reference genomes. **A** and **C**: Geographic distribution of accession clusters identified by kinship and hierarchical clustering (K = 5) on a map of northwestern South America using LA1670 (**A**) and LA1246 (**C**). **B** and **D**: PCA based on SNPs called from LA1670 (**B**) and LA1246 (**D**), with clusters overlaid. **E** and **F**: Mean annual surface air temperature (**E**) and precipitation (**F**) at sampling sites (1981-2010 period), projected onto the PCA based on LA1246. Corresponding results for LA1670 are shown in S4A and S4B Fig. Geographic origin of accessions was mapped onto both geographic (**A**) and PCA (**B**) plots to illustrate spatial distribution. The geographical distribution of the panel was plotted using passport data (S2 Table) and the ***worldmap()*** function from the *rnaturalearth* [47] R package. Basemap data (country boundaries) were obtained from Natural Earth: https://www.naturalearthdata.com/downloads/. Terms of use: https://www.naturalearthdata.com/about/terms-of-use/.

**Fig 2:**
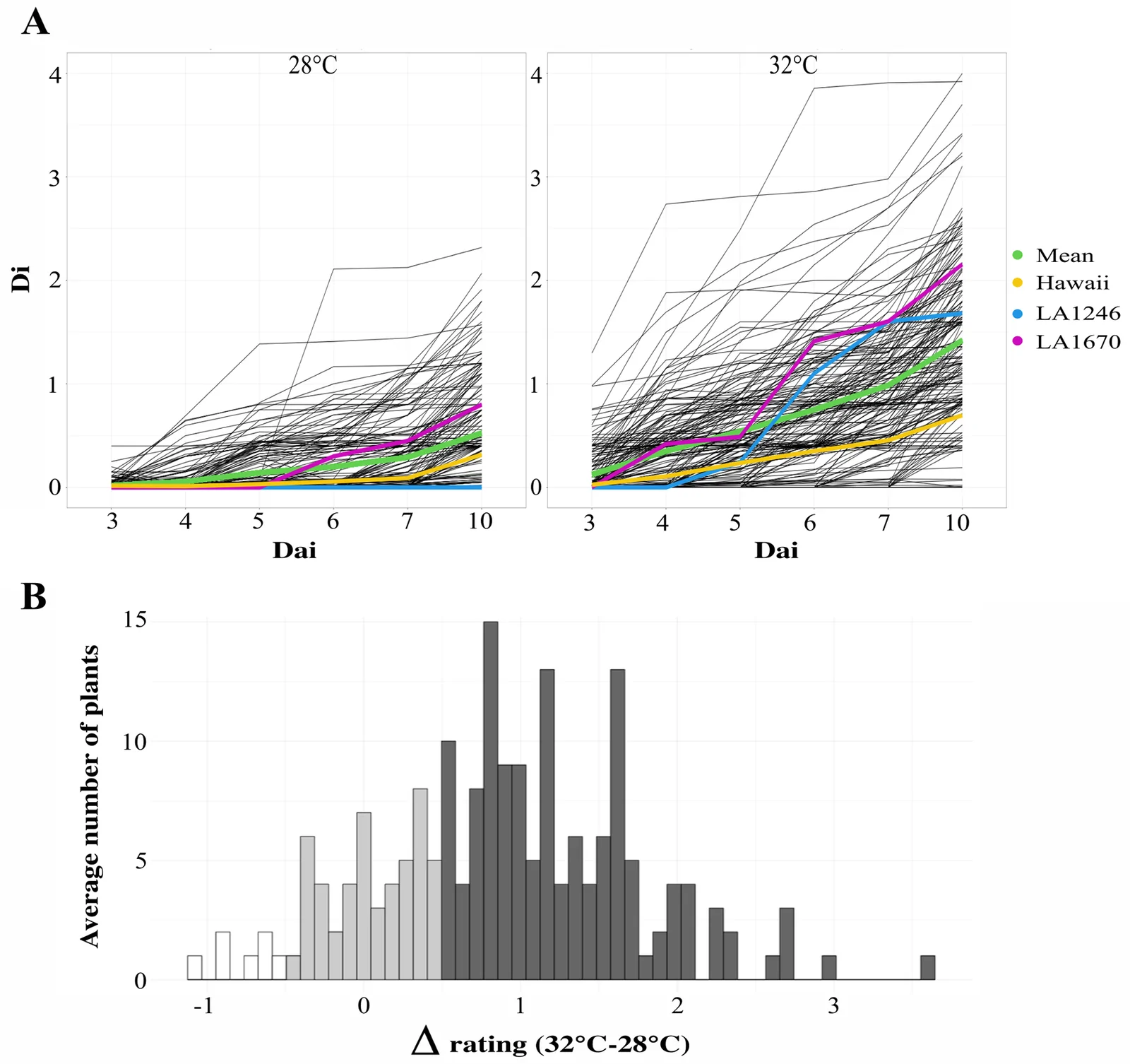
Exploration of the phenotypic response of the panel following infection by *R. pseudosolanacearum* at 28 °C and 32 °C. **A**: Kinetics of the response of panel accessions to *R. pseudosolanacearum* at 28 °C (left) and 32 °C (right). The y-axis represents LS-means of disease index (Di; 0-4), over days after inoculation (Dai). Each line represents the disease progression curve of one accession. Mean panel response is shown in green, with reference accessions ‘Hawaii 7996’ (yellow), LA1246 (blue) and LA1670 (purple). **B**: Difference in disease index between 32 °C and 28 °C at 10 Dai (ΔDi = 32 °C-28 °C). Values represent LS-means per accession. Accessions are grouped and differentially colored according to ΔDi. Dark gray: accessions more susceptible at 32 °C (ΔDi > 0.5); light gray: accessions with similar response at both temperatures (|ΔDi| < 0.5); white: accessions less susceptible at 32 °C (ΔDi < -0.5).

Most accessions of the panel originate from Peru and Ecuador, the native regions of *S. pimpinellifolium* and *S. lycopersicum* var. *cerasiforme*, with 73% from Peru and 14% from Ecuador. The *S. lycopersicum* var. *cerasiforme* accessions originated from Central America and from the western side of the Andes (Fig 1A and 1B). Some *S. pimpinellifolium* accessions were also found in this area, but most were sampled from the eastern side, either in the mountainous areas of southern Ecuador/northern Peru or in the coastal valley near the Pacific Ocean in southern Peru (Fig 1A and 1B). Probably due to this distribution on the slopes of the mountains, the accessions were collected at different altitudes with the majority (45%) found below 500 m. 14.3% found in a range of altitudes between 500 m and 1,000 m and finally 5.8% above 1,000 m. As Blanca et al. [46] showed that genetic diversity in their smaller panel of S*. pimpinellifolium* accessions was structured into two clusters, we selected two representative accessions from each cluster as internal references for HiFi sequencing. LA1670 and LA1246 were chosen not only for originating from habitats with contrasting climates, as determined from their GPS coordinates [48] (S1 Table, S1 Fig), but also for exhibiting differential responses to *R. pseudosolanacearum* inoculation at 28 °C and 32 °C (Fig 2A).

### Genomic data and comparative analysis of reference genomes

**Fig 3:**
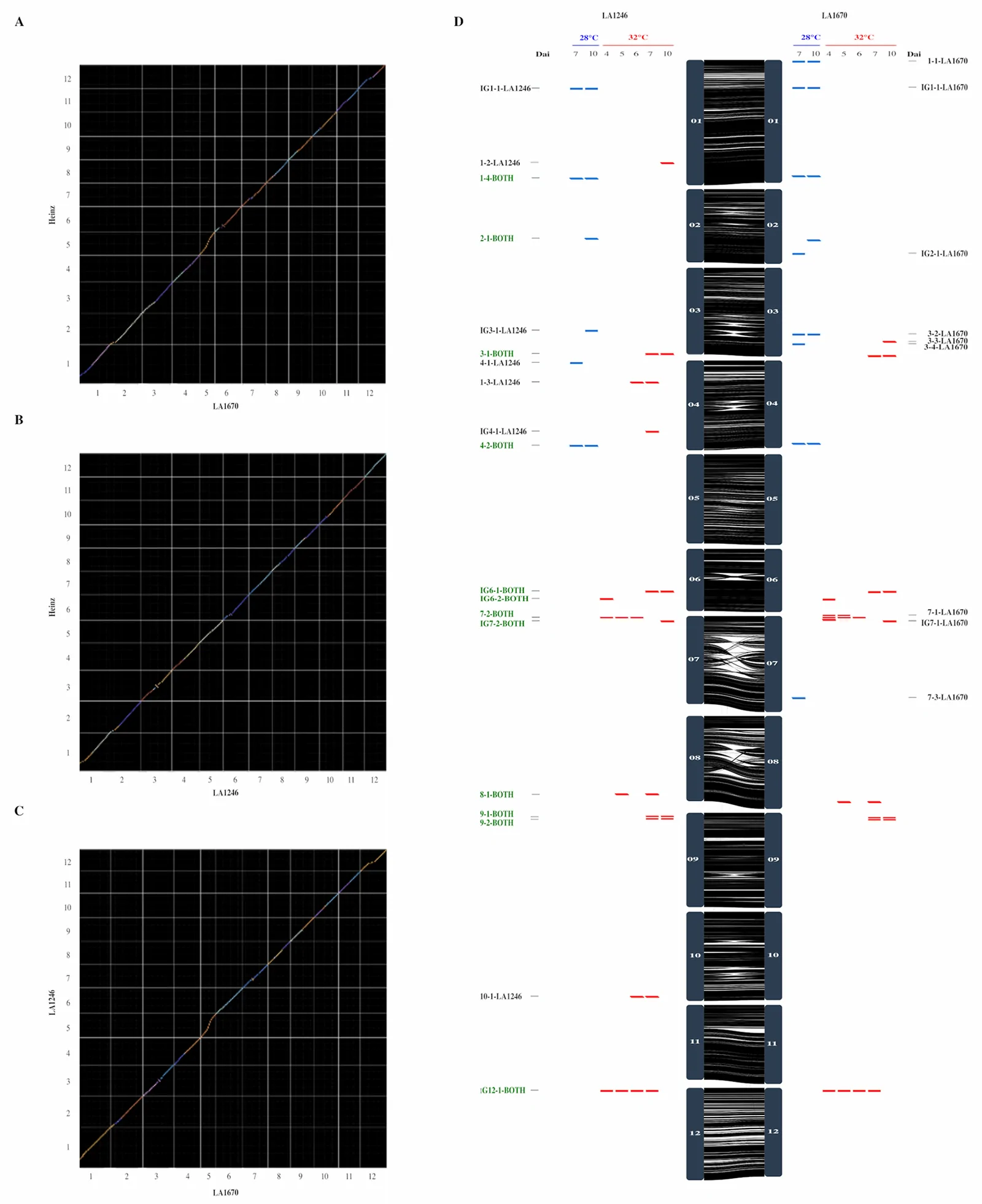
Collinearity between LA1246 and LA1670 genomes and QTLs detected either at 28 °C or 32 °C. **A** and **B**: Synteny anchor BLAST hits colored by block ID between Heinz (y-axis; 25,829 genes with BLAST hits, ITAG4.1 [50]) and LA1670 (x-axis; 25,829 genes) (**A**) or LA1246 (x-axis; 25,829 genes) (**B**). **C**: Synteny anchor BLAST hits colored by block ID between LA1246 (y-axis; 34,134 genes) and LA1670 (x-axis; 34,135 genes). Chromosome position is shown by gene rank order. Gridlines indicate every 1,000 genes. **D**: Global overview of collinearity between LA1246 and LA1670 genomes. QTLs specific at 28 °C or 32 °C are indicated. QTLs unique to LA1246 are shown on the right, those unique to LA1670 on the left, and shared QTLs are highlighted in green. Analyses, localization of QTLs and visualization were performed using GENESPACE [49].

Reference genomes were sequenced using PacBio technology and reached a depth of 82x for LA1246 and 80x for LA1670 (S2 and S3 Tables). The high sequencing depth obtained supports the accuracy of both reference genomes. Interestingly, genome assembly highlighted chromosomal rearrangements between the two reference genomes, especially on chromosomes 6, 7 and 8 (Fig 3A and 3B). The final assembled genome size was 815.7 Mb for LA1670 and 808.6 Mb for LA1246. Gene prediction revealed 46,202 (LA1670) and 45,746 (LA1246) genes (S2 and S3 Tables). Illumina sequencing of the panel revealed a depth of between 15x and 25x for raw sequenced data which varied among accessions. For SNP (Table 1, filter A), the mean coverage at a locus was between 13.66x (LA1246) and 13.81x (LA1670) (S4 and S5 Tables).

**Table 1:** Number of SNPs in matrices per reference genome after sequential filtering and their use in downstream analyses.

Filter used	Reference Genome (number of SNP)		Analyses
	LA1670	LA1246	
A	58 353 015	58 606 561	
A + biallelic	47 430 644	47 486 684	
A + B	25 622 125	25 929 267	
A + B + C	13 541 872	14 551 270	PCA
A + B + C + D	1 836 148	2 567 085	Kinship
A + B + C + E	3 728 946	3 646 481	GWAS SNPs matrix

Whole-genome dot plot comparisons were performed between the *S. lycopersicum* ‘Heinz’ reference genome [50] and those of LA1670 and LA1246, as well as between LA1670 and LA1246 (Fig 3A). All comparisons showed a nearly continuous main diagonal, indicating strong chromosome-level collinearity. Minor deviations and local breaks in the diagonal (Heinz - LA1670: chromosomes 2, 5, 12; Heinz - LA1246: chromosomes 1, 2, 3; LA1246 - LA1670: chromosomes 5, 12) suggest limited structural variation, likely reflecting small-scale rearrangements and/or local size differences (Figs 3B, S2). Consistent with the larger genome sizes of LA1670 and LA1246 relative to Heinz, these local expansions could correspond to repetitive or intergenic regions, as previously reported [51]. Orthogroup analysis performed between LA1246 and LA1670 (S6 and S7 Tables) classifyed genes according to their presence/absence and copy number variations (CNVs) across genomes (0–1, 1–0, 1–1, 0-n, n-0, 1-n, n-1, n-n). A total of 34,801 1–1 orthologs were identified, confirming a high level of gene conservation. Most differences between genomes were associated with CNVs, (1-n expansion in LA1670; n-1 expansion in LA1246) or presence/absence (0–1, 1–0), in a limited set of orthogroups. Gene ontology enrichment analyses of presence-absence orthogroups (S7 Table) revealed contrasting functional signatures between genomes. Orthogroups specific to LA1670 were strongly enriched for genes involved in chromosome segregation, mitotic division and cell cycle processes, whereas orthogroups specific to the LA1246 genome were predominantly associated with transcriptional regulation and metabolic processes. The number of specific orthogroups was slightly higher in LA1246 (2,953) than in LA1670 (2,606) corresponding to a 0.8 ratio, suggesting relatively more gene loss or contraction in LA1670. Notably, orthogroups with CNVs corresponding to genes involved in defense responses and stimulus perception were significantly overrepresented, consistent with the rapid evolution of these gene families often located in repeat-rich genomic regions as previously demonstrated [51].

Reference genomes were used to call SNPs obtained by panel sequencing. We assessed the gene SNP coverage by using filtered GWAS SNP matrices (Table 1; filters A, B, C + E). Results indicated that at least one SNP was found in 80% of genes with, on average, 9.6 SNPs/gene whichever reference genome was chosen. This coverage varied between 7.5 and 13.2 SNPs/gene depending on the chromosome (S3 Fig).

### A continuous population structure shaped by geographic and environmental gradients and a LD suitable for GWAS

Population structure was studied through principal component analysis (PCA) using SNPs called from the two reference genomes. (Table 1; filters A - C). The first two PCA axes (Fig 1), explaining cumulatively around 10% of the genetic variation (LA1670: Dim1 6.53% - Dim2 4.07%; LA1246: Dim1 5.53% - Dim2 3.82%) revealed the limited global genetic structure within the panel, with individuals distributed along an arch pattern indicative of a continuous gradient. To determine the information that could be described by both axes, we first report manually the provinces of origin on the PCA results and on a map of northwestern South America to visualize their geographical distribution (Fig 1A and 1B). We also report on PCA results obtained with both reference genomes i) species distribution (S4C and S4D Fig), ii) mean annual surface air temperature (Figs 1E, S4A) and mean annual precipitation (Figs 1F, S4B) at the sampling site, when available, during the 1981–2010 period. The spatial distribution of individuals along the PCA axes seemed to reflect a geographic east-west gradient on the first axis, reflecting genetic variation from populations along the Pacific coast, across the western slopes of the Andes, to more inland regions of central Peru. The second axis mostly captured both a south-north geographic gradient and climatic variation, ranging from colder and drier to warmer and more humid conditions. These results support a scenario of continuous genetic variation shaped by both geographic and environmental factors. Hierarchical clustering using *hclust* applied to the kinship-derived distance matrix identified five groups (K = 5). When projected onto PCA results and on a map of northwestern South America (Fig 1A and 1D), clusters aligned with the main genetic gradient and corresponded to the geographic distribution of accessions. Pairwise F ST estimates revealed moderate to high genetic differentiation among clusters, confirming that these groups reflect non-random population structure. Overall, genetic variation appears structured along continuous geographic and environmental gradients rather than discrete populations. Using LA1246 as an alternative reference genome produced slightly different hierarchical clusters, despite high overall genome similarity (Fig 1C and 1D).

The extent of LD was investigated using the filtered GWAS matrix (Table 1; filters A, B, C + E); its half-decay was calculated per chromosome and is of the order of a few kb for most chromosomes, with the exception of chromosomes 5, 6 and 8 for which LD decay was lower (S5 Fig, S8 Table). Specifically, LD visualization on these latter chromosomes showed large blocks of LD, located in central chromosomal regions, and low LD levels in telomeric regions (S6 Fig).

### Panel responses to R. pseudosolanacearum reveal three temperature-dependent profiles

Panel accessions were phenotyped at 28 °C and 32 °C for bacterial wilt disease severity for ten days following pathogen inoculation (Fig 2). Overall, the 189 accessions were on average more susceptible at 32 °C than at 28 °C. At 28 °C, the two reference accessions displayed contrasting responses to *R. pseudosolanacearum*, with LA1246 remaining symptomless. This difference disappeared at 32 °C, where both accessions developed wilting symptoms. The cultivar ‘Hawaii 7996’, included as a known tolerant control at 28 °C, began to wilt at the end of the infection kinetic at 32 °C (Fig 2A). At 28 °C, no genetic variation was detected from 3 to 6 dai, whereas *H²* reached 0.19 and 0.24 at 7 and 10 dai, respectively (Table 2). In contrast, at 32 °C, genetic variation was detected at all time points except at 3 dai, with *H²* values fluctuating from 0.11 to 0.29. At both temperatures, *H²* was the highest at 10 Dai, with slightly higher values at 32 °C. Notably, at 32 °C, *H²* was already of a similar magnitude at 4 dai, suggesting greater genetic variability both at early stages of the plant-pathogen interaction and during disease progression. Although experiments were conducted under controlled conditions, the dynamic nature of disease development may have led to disparity in symptom onset between blocks phenotyped at different times. Such temporal shifts may reduce heritability estimates when blocks are analysed jointly. However, the higher heritability observed along the infection kinetic at 32 °C may reflect a stronger and more synchronous expression of plant responses, facilitating QTL detection.

**Table 2:** Natural variation among the accessions of the panel for disease index at 28 °C and 32 °C.

Temperature	Model terms	Dai 3		Dai 4		Dai 5		Dai 6		Dai 7		Dai 10	
		F/LRT	p-value	F/LRT	p-value	F/LRT	p-value	F/LRT	p-value	F/LRT	p-value	F/LRT	p-value
28 °C	Block	4.87	0.0273	12.14	0.0005	15.7	0.0001	21.83	<0.0001	30.21	<0.0001	92.38	<0.0001
	Accession	0.91	0.7710	0.97	0.5930	0.95	0.6700	1.11	0.1800	1.3	0.0087	1.49	0.0001
	Interaction	1.38	0.2400	116.36	<0.0001	37.85	<0.0001	30.19	<0.0001	16.43	<0.0001	25.98	<0.0001
	*H* ^*2*^	0.000		0.000		0.000		0.000		0.182		0.242	
32 °C	Block	15.07	0.0001	65.66	<0.0001	91.29	<0.0001	123.82	<0.0001	121.65	<0.0001	119.88	<0.0001
	Accession	1.07	0.2810	1.48	0.0002	1.33	0.0052	1.38	0.0018	1.35	0.0037	1.64	<0.0001
	Interaction	44.61	0.0000	9.62	0.0019	8.77	0.0031	16.93	0.0039	15.9	<0.0001	12.75	0.0004
	*H* ^*2*^	0.006		0.247		0.123		0.111		0.111		0.293	

Based on adjusted means disease severity ratings, a cross-temperature genetic Pearson correlation was calculated, at 10 dai between 28 °C and 32 °C, time point at which *H²* was the highest. The low correlation (r = 0.3) indicates that the genetic control of disease progression is only partially shared between the two temperatures, suggesting a substantial genotype-by-temperature interaction and a flexible genetic architecture underlying the response of accessions to inoculation with *R. pseudosolanacearum* GMI1000 strain. Several accessions remained symptomless until the end of the experiment, at both temperatures (Di < 0.2) (S7 Fig), suggesting the presence of strong resistance mechanisms in the panel. To explore temperature-dependent responses, reaction norms were plotted at 10 Dai between both temperatures, revealing four different profiles (S7 Fig). Finally, by comparing the adjusted mean disease severity ratings, three temperature-dependent response profiles were defined: (1) accessions that were more susceptible at 32 °C than at 28 °C (133 out of 189 accessions), (2) accessions displaying similar responses at both temperatures (49 out of 189), and (3) accessions showing lower susceptibility at 32 °C (7 out of 189) (Fig 2B).

### GWAS and post-GWAS analyses identify 44 candidate genes putatively involved in bacterial wilt defense response at 28 °C and 32 °C

GWAS results are presented in (Tables 3 and S9, S8 Fig). After QTL filtering, 21 “strict candidate genes” corresponding to 18 QTLs were selected. QTL intervals did not exceed a few kb, with most loci underlying a single candidate gene, indicating near gene-level resolution. Exceptions included QTL *1–3-LA1246* (12 kb, high gene density) and QTL *1–4-BOTH*, which encompassed two genes; however, the most significant SNPs in both references (SpimpiLA1670Chr01_88223852_T for LA1670 and SpimpiLA1246Chr01_89956087_T for LA1246) mapped to an exon of the *Solyc01g105270* homolog. Considering “intergenic candidate genes” (Table 3) added 23 genes related to 10 oher QTLs. Although these did not meet proximity criteria, some were recurrently detected along the kinetic of infection, notably QTL *IG12–1-BOTH* identified over four consecutive days (**S8 Fig**), the maximum observed.

**Table 3:** Synthetic overview of the QTL and corresponding candidate genes identified in this study at 28 °C (A) and 32 °C (B).

A								
QTL name (*a*)	Reference genomes (*b*)	Dai of detection LA1246/ LA1670 (*c*)	*S. lycopersicum* homolog (*d*)	Expression pattern (*e*)	*A. thaliana* ortholog (*f*)	Expression pattern (*g*)	Functional category (*h*)	References
1-1-LA1670	LA1246*/LA1670	7/ 7, 10	*Solyc01g006660* Subtilisin-like serine protease	Roots; leaves (*S.pimpinellifolium*) - nucleus	*At1g20160* ATSBT5.2,CRSP,S CO2 RESPONSE SECRETED PROTEASE; Subtilisin-like serine endopeptidase family protein	Roots, stem, extracellular - endoplasmic reticulum, defense reponse to bacteria	4	76,77
3-2-LA1670	LA1246*/LA1670	10/ 7, 10	*Solyc03g093130* xyloglucan-modifying enzyme	Mature green, breaker fruits, roots	Multigene family	na	na	
3-4-LA1670	LA1246*/LA1670	7/ 7	*Solyc03g019730* Sumo activating enzyme 1b, Molybdenum cofactor biosynthesis	Unopened flower buds, roots, leaves - mitochondrion	*At5g50580, At5g50680* AT-SAE1–2, SAE1B, sumo-activating enzyme 1B	Seeds - cytosol	4	
4-1-LA1246	LA1246/LA1670*	7/ 7	*Solyc04g008310.* UDP-glucuronosyl/UDP-glucosyltransferase	Breaker fruits - cytosol	*At2g15490 UDP-glycosyltransferase 73B4*	Seeds, roots - cytosol, cell wall, response to biotic and abiotic stresses	1	66
7-3-LA1670	LA1246*/LA1670	7/ 7	*Solyc07g044730* 3-hydroxyisobutyryl- CoA hydrolase-like protein	Opened flowers	*At1g06550 Multigene family*	na	5	
1-4-BOTH	LA1246/LA1670	7,10/ 7,10	*Solyc01g105260* Integral membrane protein Protein of unknown function DUF125, vacuolar iron transporter homolog 4-like	Roots - plasma membrane	na	na	1	
1-4-BOTH	LA1246/LA1670	7,10/ 7,10	*Solyc01g105270* LeTH2 tobamovirus multiplication 1 homolog 2	Roots,fruits, flowers	na	na	1	68
2-1-BOTH	LA1246/LA1670	10/ 10	*Solyc02g067460* Mitochondrial outer membrane protein porin	Young fruits, roots, *leaves (S.pimpinellifolium*) - mitochondrion	Multigene family	na	5	
4-2-BOTH	LA1246/LA1670	7,10/ 7,10	*Solyc04g078590* Receptor kinase; serine/threonine kinase	Young, fruits - plasma membrane	Multigene family	na	3	25
IG1–1-LA1246	LA1246/LA1670*	7,10/ 7,10	na	na	na	na	na	
IG1–1-LA1670	LA124*/LA1670	7,10/ 7,10	*Solyc01g016350* unknown protein	Extracellular	na	na	5	
IG2–1-LA1670	LA1670	na/ 7	*Solyc02g084670*	Unopened flower buds - mitochondrion	Multigene family	na	5	
IG2–1-LA1670	LA1670	na/ 7	*Solyc02g084680* Protein SHI RELATED SEQUENCE 5	Flowers, roots, leaves	Multigene family	na	5	
IG3–1-LA1246	LA1246	10/ na	na	na	*At4g12560* (?)	na	5	
IG3–1-LA1246	LA1246	10/ na	*Solyc03g093770* histone-lysine methyltransferase	Leaves	na	na	5	
**B**								
**QTL name (*a*)**	**Reference genomes (*b*)**	**Dai of detection LA1246/ LA1670 (*c*)**	***S. lycopersicum* homolog (*d*)**	**Expression pattern *(e*)**	***A. thaliana* ortholog (*f*)**	**Expression pattern (*g*)**	**Functional category (*h*)**	**References**
1-2-LA1246	LA1246/LA1670*	10/ 10	*Solyc01g086890* Splicing factor 4-like protein G-patch	Roots, fruits, flowers	*At3g52120* CIS1, CRY2 interacting splicing factor 1, regulate thermosensory flowering via FLM alternative splicing.	Everywhere - nucleus, cytosol	5	
1-3-LA1246	LA1246/LA1670*	6, 7/ 6	*Solyc01g100650* NHL repeat-containing protein 2-like	Leaves - mitochondrion	*At4g35090* (?) Multigene family	Everywhere - plastids; response to pathogen infection	5	
1-3-LA1246	LA1246/LA1670*	6, 7/ 6	*Solyc01g100630* Catalase	Leaves, roots - peroxisome	*At4g35090* (?) Multigene family	Everywhere - peroxysome, mitochondrion, response to infection	2	
1-3-LA1246	LA1246/LA1670*	6, 7/ 6	*Solyc01g100640* Catalase	Breaker fruits - peroxisome	*At4g35090* (?) Multigene family	Everywhere - peroxysome, mitochondrion, response to infection	2	
3-3-LA1670	LA1246*/ LA1670	10/ 10	*Solyc03g097470 3*-Beta-ketoacyl-acyl carrier protein synthase III (FabH)	Breaker fruits, mitochondrion	*At1g62640* 3-ketoacyl-acyl carrier protein synthase III	Everywhere - response to infections	5	65
7-1-LA1670	LA1670	na/ 4, 5	*Solyc07g005040, Solyc00g257110* Plasma membrane ATPase	Flowers	Multigene family	na	5	
10-1-LA1246	LA1246/LA1670*	6, 7/ 7	*Solyc10g081260* Multidrug resistance protein mdtK Protein Multi antimicrobial extrusion protein MatE	Young and breaker fruits - plasma membrane	*At5g17700* MATE efflux family protein	Roots, petals - plasma membrane - response to infections	1	65
3-1-BOTH	LA1246/LA1670	7, 10/ 7, 10	*Solyc03g123790* Vacuolar calcium cation/proton exchanger	Roots, mature green fruits, plasma membrane	*At1g55730 (?)* Multigene family	na	2	
7-2-BOTH	LA1246/LA1670	4, 5, 6/ 4, 5, 6	na	na	na	na		
8-1-BOTH	LA1246/LA1670	5, 7/ 5, 7	na	na	na	na		
9-1-BOTH	LA1246/LA1670	7, 10/ 7, 10	*Solyc09g009700* GDSL esterase/lipase	Roots, opened flowers, extracellular	*At5g03610* GGL25 - GDSL-motif esterase/acyltransferase/lipase	Stem, embryo, primary root - endoplamsic reticulum, extracellular - response to pathogen and nitrogen	1	67
9-2-BOTH	LA1246/LA1670	7, 10/ 7, 10	*Solyc09g011630* Glutation-S-transferase	Roots, cytosol	*At2g29420 (?)* Glutathione S-transferase 25, Glutathione S-transferase TAU 7, GST25, GSTU7	Roots, petals - Response to hormones and infection	4	
9-2-BOTH	LA1246/LA1670	7, 10/ 7, 10	*Solyc09g011640* Glutation-S-transferase	Roots, cytosol	*At2g29420* Glutathione S-transferase 25, Glutathione S-transferase TAU 7, GST25, GSTU7	Roots, petals - Response to hormones and infection	4	
IG4–1-LA1246	LA1246/LA1670*	7/ 7	na	na	na	na		
IG4–1-LA1246	LA1246/LA1670*	7/ 7	Multigene family	na	Multigene family	na		
IG4–1-LA1246	LA1246/LA1670*	7/ 7	*Solyc00g028960* Glutaredoxin family protein	Leaves (S.pimpinellifolium)	Multigene family	na	3	
IG6–1-BOTH	LA1246/LA1670	7, 10/ 7, 10	Multigene family	na	Multigene family	na		
IG6–1-BOTH	LA1246/LA1670	7, 10/ 7, 10	*Solyc06g051510* Protein kinase superfamily protein	Roots, mature green fruits, nucleus	Multigene family	na	3	
IG6–2-BOTH	LA1246/LA1670	4/ 4	*Solyc06g065060* FAD-binding domain-containing protein	Mature green fruits, vacuole	Multigene family	na	5	
IG6–2-BOTH	LA1246/LA1670	4/ 4	*Solyc06g065070* FAD-binding domain-containing protein	Mature green fruits, vacuole	Multigene family	na	5	
IG7–1-LA1670	LA1246*/LA1670	4/ 4	*Solyc07g007600* vacuolar-type H + -pyrophosphatase	Roots, mature grenn fruits, leaves (S. pimpinellifolium), nucleus	*At1g15690*	Everywhere - vacuole and plasma membrane - response to some infections	5	
IG7–1-LA1670	LA1246*/LA1670	4/ 4	*Solyc07g007610* At2G21500 protein (Fragment)	Opened and unopened flowers, nucleus	na	na		
IG7–2-BOTH	LA1246/LA1670	10/ 10	*Solyc07g007940* Small nuclear RNA activating complex (SNAPc), subunit SNAP43 protein	Roots, immature fruits, cytosol	*At3g53270*	Everywhere - cytosol, nucleus - response to infections	5	65
IG7–2-BOTH	LA1246/LA1670	10/ 10	na	na	na	na		
IG12–1-BOTH	LA1246/LA1670	4, 5, 6, 7/ 4, 5, 6, 7	*Solyc12g008950* DEAD-box ATP-dependent RNA helicase	Roots, young and breaker fruits - nucleus	*At1g72690 (?)*	Nucleus; response to infections	1	
**IG12–1-BOTH**	**LA1246/LA1670**	**4, 5, 6, 7/ 4, 5, 6, 7**	***Solyc12g008960* Calmodulin-binding protein**	**Roots - nucleus**	***At4g33050 (?)***	**Everywhere - cytosol, mitochondrion, nucleus - response to some infections**	**1**	

(***a***) name of the QTL. (***b***) The reference genome(s) on which the QTL was detected. An asterisk (*) stipulates that the QTL underlying the candidate gene was detected with one reference genome and was below the threshold p-value of detection with the other reference genome, even if an association peak was observed (see **S8A**-**S8F** Fig for details). (***c***) Dai at which QTL were detected with LA1246 or LA1670. (***d***) *S.lycopersicum* homologous gene ID and its related annotation found for the ‘Heinz’ reference [50]. (***e***) Summary of known gene expression patterns information in organs - cell - experimental conditions, retrieved from eplant tomato visualization tools (https://bar.utoronto.ca/eplant_tomato/). (*f*) Corresponding *A. thaliana* orthologous gene ID and its related annotation when available; (?) corresponds to genes with low-confidence BLAST results. (***g***) Summary of known gene expression patterns information in organs - cell - experiment conditions retrieved from eplant *A. thaliana* visualization tools (https://bar.utoronto.ca/eplant/). Order of organs and subcellular compartments indicate the range of upregulation, the first being the one in which the gene is the most highly overexpressed. (***h***) Functional category: 1-immunity, 2-calcium signaling related, 3-RLKs, 4- may be involved in plant defense, 5-link to be demonstrated. na: not available.

### GWAS using two reference genomes help to detect QTLs

**Fig 4:**
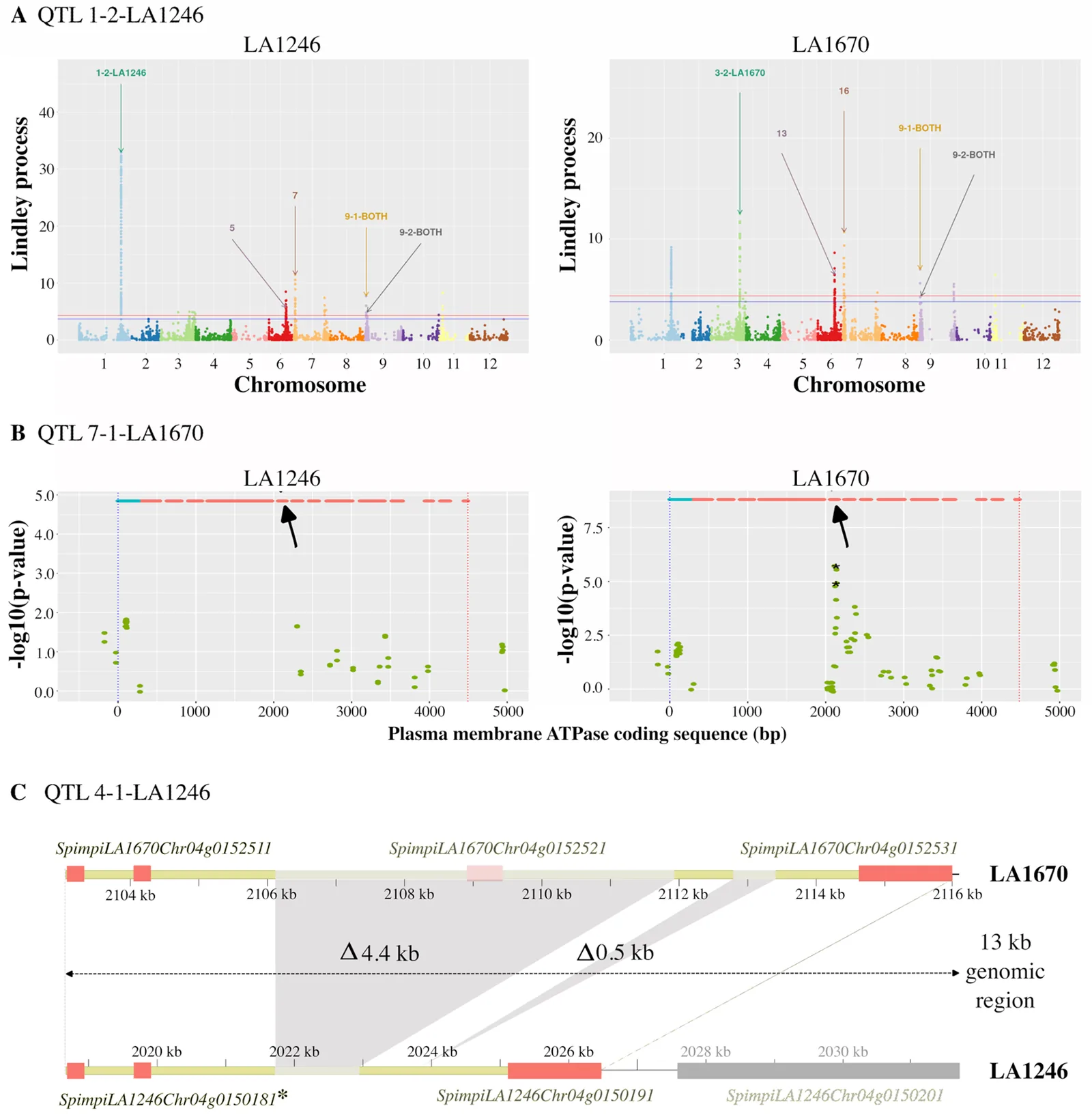
Three different cases for which the use of two reference genomes improved QTL detection. **A**: Effect of the reference genome on QTL detection resulting from the selection criteria used. GWAS results at 32 °C and 10 Dai using LA1246 (left) and LA1670 (right) reference genomes. The light-blue peak on chromosome 1 corresponds to QTL *1-2-LA1246*, detected in LA1246 but not in LA1670 due to differences in the detection p-value threshold (* in Table 3). The x-axis represents marker chromosomal positions and the y-axis shows local score values. The maximal (red line) and minimal (blue line) detection thresholds for QTLs with the local score method are indicated. **B**: Example of SNP detection failure using an alternative reference genome. QTL *7-1-LA1670* is detected using the LA1670 (right) but not identified using LA1246 (left) due to the absence of SNP detection in the exon. SNPs (dots) are positioned along the gene sequence, whose structure (exon in red, 3’ UTR in blue) is shown at the top of each graph. Arrows indicate the exon where the QTL is detected in LA1670. The y-axis represents -log10 (p-values). Random noise was added to the x-axis for vizualization purposes for all p-values in both conditions. * indicate the most significant SNPs. **C**: Structural variation between the LA1670 and LA1246 reference genomes. Two indels (genomic regions delimited by dotted lines) that may explain the absence of detection of QTL *4-1-LA1246* counterparts in LA1670. Conserved genomic regions are shown in light green and divergent regions in light grey.

Among the 21 strict candidates, only one was found specific to LA1670, nine were detected in both reference genomes, while 11 showed significant association in one genome with an association peak slightly below the detection threshold in the other (indicated by a * in Tables 3 and S9), and were thus considered nearly shared. Differences in QTL detection can be explained by three main factors. First, statistical threshold effects led to marginal QTLs being retained in only one reference, as illustrated by QTL *1–2-LA1246* (Tables 3 and S9, Fig 4A). The GWAS model used was based on a statistical test using both kinship and population structure. Despite strong correlations between kinship matrices (>0.9), differences at the whole genome level may impact its coverage and the ability to detect associations. Second, differences in variant calling at the locus level due to sequence variations and local sequence divergence between reference genomes. This is illustrated by the QTL *7–1-LA1670*, underlying a gene encoding an APT8 Plasma membrane-like protein where SNP coverage differs depending on the reference genome used (Fig 4B). In LA1246, the exon containing the top SNP is not covered by any variant call, preventing identification. Third, structural variation between genomes contributed to differential detection. BLAST comparisons revealed indels and inversions affecting QTL regions. For example, QTL *4–1-LA1246* underlying a gene encoding a potential UDP-glycosyltransferase is located near two indels in the LA1246 genome that may have affected its detection in LA1670 (Fig 4C). Other genomic inversions were detected near QTL *3–2-LA1670*, and near QTL *IG3–1-LA1246*, and a 200 kb indel separates the two genes underlying QTL *IG8–1-LA1670*. Additionally, BLAST analysis revealed that the gene closest to QTL *IG2–1-LA1670*, annotated as a putative serine/threonine phosphatase in LA1670, is absent in the LA1246 genome.

### GWAS highlight temperature and time-dependent biologically relevant candidate genes

**Fig 5:**
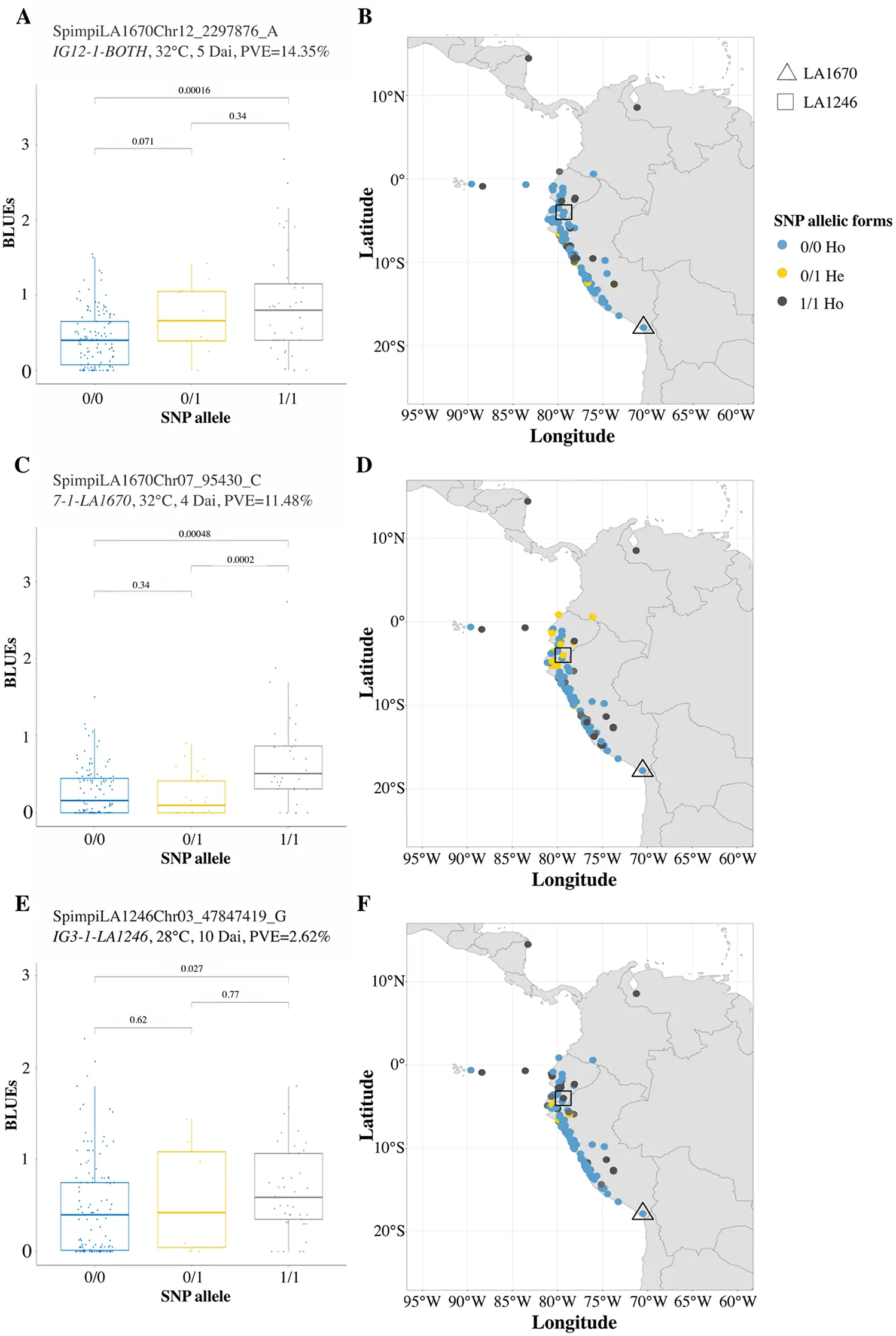
Putative biome-dependent adaptation of accessions associated with allelic effects of three selected top SNPs underlying three distinct QTLs. For each SNP, allelic effects, phenotypic variance explained, and geographic distribution of alleles on a map of northwestern South America. **A**: SpimpiLA1670Chr12_2297876_A (QTL *IG12-1-BOTH*; at 32 °C, 5 Dai). **B**: SpimpiLA1670Chr07_95430_C (QTL *7-1-LA1670*; at 32 °C, 4 Dai). **C**: SpimpiLA1246Chr03_47847419_G (QTL *IG3-1-LA1246*, 28 °C, 10 Dai). The geographical distribution of the panel was plotted using passport data (S2 Table) and the ***worldmap()*** function from the *rnaturalearth* [47] R package. Basemap data (country boundaries) were obtained from Natural Earth: https://www.naturalearthdata.com/downloads/. Terms of use: https://www.naturalearthdata.com/about/terms-of-use/.

Although similar numbers of QTL underlying « strict candidate genes » (8 vs 10) or « intergenic candidate genes (4 vs 6) were detected at 28 °C and 32 °C, no QTL was shared between the two conditions, indicating temperature-specific genetic control of the response. QTL detection also varied over time, with peaks appearing transiently across phenotyping days (**S8 Fig**). Most QTLs were detected over one or two consecutive days, with few persisting longer (up to four days only for *IG12–1-BOTH* at 32 °C), suggesting dynamic defense response at elevated temperature. At 28 °C, most QTLs were detected at the end of the kinetic (8/12 at 10 Dai), whereas QTLs detected at 32°C were distributed throughout the infection process. Three detection profiles could be defined: QTLs detected mainly at the beginning (3/16), throughout (3/16), or at the end (10/16) of the infection process. To test the hypothesis that defense - response - associated loci may be structure by environmental conditions, we examine allelic effects and phenottypic variance explained by three top SNPs represenative of three QTLs characterized by their temporal stability, reference genome specificity and temperature-dependent association patterns (Fig 5). The SNP SpimpiLA1670Chr12_2297876_A (QTL *IG12–1-BOTH*; detected at 32 °C, 5 Dai; Fig 5A) showed no clear association between allele state and geographic origin of accessions, suggesting an absence of local adaptation at this locus. In contrast, the two reference-specific SNPs SpimpiLA1670Chr07_95430_C (QTL *7–1-LA1670*; identified at 32 °C, 4 Dai; Fig 5B) and SpimpiLA1246Chr03_47847419_G (QTL *IG3–1-LA1246*; detected at 28 °C, 10 Dai; Fig 5C) displayed structured distributions with specific genotypic states at these SNPs predominantly observed in accessions originating from contrasting climatic regions.

To further explore the biological relevance of the strongest associations, homology analyses were performed and expression profiles were investigated for « strict candidate genes » (Tables 3 and S9). Out of the 21 « strict candidate genes », information relative to their homolog expression profile in *S. lycopersicum* was retrieved for 19 genes, with 12 of them exhibiting upregulated expression in roots (9 strongly regulated), but also in other organs and different cell types. Five of them have already been described in previous studies (*Solyc01g006660, Solyc01g105270, Solyc04g008310, Solyc09g009700, Solyc10g081260*) as associated with plant immunity. Only eight high-confidence BLAST *A. thaliana* orthologs were identified, reflecting phylogenetic distance. Among them, five were also found to be expressed in roots and six had their expression regulated under biotic stress. Although functional data remain limited for several genes, overall patterns indicated enrichment in root-expressed, defense-related candidates responsive to pathogen challenge. Regarding “intergenic candidate genes,” six genes located near five QTLs, including *IG12–1-BOTH* detected between 4 and 7 Dai, are also reported to be expressed in the roots.

## Discussion

Initially, our goal was not only to identify elevated temperature resistance but also to uncover genetic mechanisms that remain stable across temperatures, as many loci effective under standard conditions are negatively affected by heat stress [3] and some can be detected through GWAS [21,28,29]. To this end, we implemented a GWAS approach using a selected panel of wild tomatoes, with a higher representation of *S. pimpinellifolium* accessions, sequenced on this occasion, and including two internal reference genomes. To investigate the stability of resistance mechanisms to *R. pseudosolanacearum* effective at elevated temperatures and identify novel mechanisms, we studied the tomato/*R. solanacearum* pathosystem under two contrasted temperature conditions. While 28 °C is commonly used for phenotyping tomato genotypes in controlled conditions [52], we included 32 °C to represent an extreme warming scenario (SSP5-8.5; 4–5 °C increase by the end of the century; [4]) and because this temperature has been reported to compromise the defense response of the most tolerant Hawaii 7996 cultivar in the field [42].

### High temperature influences the genetic architecture of tomato defense responses

This study supports a strong impact of temperature on the genetic architecture of the plant defense response. The absence of shared QTLs between the two temperature conditions indicates that most loci are **temperature-specific rather than temperature-stable**, suggesting that robustness of plant defense may be a rare and complex trait. This interpretation is validated by the limited number of symptomless accessions observed, and is consistent with previous studies in *A. thaliana*, where resistance responses to bacterial infection were also highly temperature-dependent [21,53], with only a single accession remaining without symptoms at both 27 °C and 30 °C [21]. It supports the view that plant immunity is dynamic, depending on the temporal expression of bacterial pathogenic determinants during the infection progress, both being sensitive to environmental conditions, particularly temperature [28,38,54]. This conclusion is reinforced by the phenotypic diversity observed within the panel at elevated temperature. Indeed, we detected increased variability in infection dynamics, earlier and stronger heritability of the response to the bacterium, and an overall shift toward higher susceptibility at 32 °C for the majority of accessions. This pattern is confirmed by the low genetic correlation between conditions, indicating distinct genetic determinisms. This contrasts markedly with prior observations in *A. thaliana*, in which response variability was greater at 27 °C whereas most accessions became uniformly susceptible at 30 °C [21,28]. Such differences suggest that wild tomato relatives may harbor a broader repertoire of adaptive responses to combined biotic and heat stress. Finally, analysing the phenotypic responses at the endpoint of the infection kinetics revealed three different groups of accessions across temperatures: those exhibiting increased susceptibility at 32 °C, those showing reduced susceptibility and those with a stable response across conditions. This diversity in reaction norms supports the existence of diverse environment-specific genetic architectures underlying combined stress responses.

### Traces of local environmental adaptation in the panel

Our results provide partial support for the hypothesis of biome-dependent adaptation of resistance mechanisms. Indeed, examination of allelic effects and phenotypic variance explained by three top SNPs corresponding to the three selected QTLs highlights contrasting geographic patterns. The QTL *IG12–1-BOTH* showed no clear association between allele distribution and geographic origin, suggesting a generalist effect whereby the locus contributes to resistance independently of the environmental or climatic context. In contrast, the LA1670-specific QTL *7–1-LA1670* exhibited a structured distribution, with heterozygous genotypes at the top SNP predominantly originating from equatorial warm and humid regions, consistent with a putative role in adaptation to high-temperature environments. The LA1246-specific QTL *IG3–1-LA1246* displayed an opposite pattern, with the resistance-associated allele mainly found in accessions originating from cooler and drier environments, reflecting distinct selective pressures across climatic gradients. These patterns suggest that local environmental conditions may have contributed to shaping resistance to *R. solanacearum*, in agreement with the distribution of RSSC phylotype II strains in Central and South America [5,55].

### A continuous diversity structured along geographic and climatic gradients

Even if the panel spans a broad geographic range, population structure analyses revealed limited genetic subdivision, raising questions about species delimitation within *S. pimpinellifolium* and closely related taxa. This pattern, already described [46], may reflect ongoing gene flow, incomplete lineage sorting, and/or the impact of domestication-related introgressions. Rather than forming discrete genetic clusters, the observed diversity appears largely continuous and structured along geographic and climatic gradients, supporting potentially adaptive variation influenced by multiple environmental factors. Notably, the clustering of ‘Hawaii 7996’ with a subset of accessions including the reference LA1246 in the PCA may reflect introgression from wild relatives, as previously proposed [56], although uneven sampling of *S. lycopersicum* accessions could also contribute to this pattern.

### Beyond a single reference: capturing structural variation with two genomes

A key methodological aspect of our study was the use of two internal reference genomes for GWAS. Most tomato GWAS used the ‘Heinz’ cultivar reference genome [57,58] whose domestication history has reduced genetic diversity, particularly in genomic regions associated with immunity [59]. Although pangenomes provide an ideal alternative for capturing structural variation [60], their construction remains computationally demanding [61]. Here, we adopted an intermediate strategy, using two reference genomes of *S. pimpinellifolium* accessions selected for their contrasting genetic and environmental backgrounds. Comparative analyses revealed high overall collinearity with the ‘Heinz’ reference and the presence of a large number of one-to-one orthologues, indicating strong genome-wide conservation. We also identified localized structural variations, including rearrangements on chromosomes 6, 7, and 8, as well as CNVs and gene presence/absence differences. In these variable regions, genes associated with cell division (LA1670) and transcriptional or metabolism regulation (LA1246) were significantly more frequent. Notably, genes related to stress responses and defense mechanisms were overrepresented in CNVs, corroborating previous observations in *S. pimpinellifolium* [62] and confirming their relevance for our study. While powerful, the multi-reference strategy introduces complications that must be taken into account, as previously reported in maize or fungi [63,64]. Some QTLs, such as QTL *7–1-LA1670*, were detected in only one reference, putatively due to variants that could not be reliably called in the homologous region of the other genome. Small differences in kinship and population structure matrices influenced marker significance, affecting QTL detection. Additionally, structural variations altered local linkage disequilibrium patterns, impacting the detection of QTLs using local score methods. Nevertheless, this strategy enabled us to capture a broader spectrum of genetic variation than a single reference, increasing the likelihood of identifying functionally relevant loci and providing a more comprehensive view of the genetic architecture underlying tomato immunity under heat stress.

### Temperature-dependent temporal deployment of resistance

Beyond these aspects, our analyses support a **dynamic and context-dependent architecture of resistance**, shaped by both temperature and infection stage. The identification of temperature-specific QTLs, combined with the identification of root-expressed candidate genes, suggests that plant responses to combined stress involve a **temporally coordinated deployment of defense mechanisms**. This is consistent with an emerging view in which pattern-triggered immunity (PTI) and effector-triggered immunity (ETI), the two layers of immune response induced upon pathogen attack in the zig-zag model [65], are interconnected and differ in their temporal dynamics [66]. Moreover, experimental evidence from the *A. thaliana* - *Pseudomonas syringae* pathosystem suggests that temperature modulates both pathogen virulence and plant immune responses. At permissive temperatures, which support optimal growth of *A. thaliana,* ETI is strongly activated, limiting bacterial proliferation. In contrast, higher temperatures tend to suppress ETI and favor PTI, leading to a defense response that is less specific but broader. These findings highlight how temperature fluctuations can shift the balance between distinct immune pathways, influencing both disease outcome and the effectiveness of plant defense [21,53]. In parallel, *R. solanacearum* infection progression from root invasion to vascular colonization was described to involve distinct host responses at each stage [54]. Together, our results suggest that temperature may impact the temporal deployment of these different immune responses.

### Interconnected pathways underlying tomato responses

At the molecular level, the identified candidate genes point toward **multiple and interconnected biological pathways**. These include classical immunity-related processes [67–72], calcium signaling components involved in stress signal integration [73–75], receptor-like kinases mediating pathogen perception [76,77], and broader metabolic and regulatory pathways such as detoxification and protein turnover [78–80]. *A final functional category contains remaining genes for which little functional information is currently available or whose link with immunity remains to be demonstrated. Our results align with previous studies that showed that QDR* does not rely on a single class of genes but rather emerges from the integration of multiple physiological processes *[*25,66*].* This complexity is likely amplified under combined stress conditions, as each specific stress combination may trigger distinct plant responses [3]. Importantly, our results also provide new insights into previously described major QTLs such as *bwr-6* and *bwr-12*, which have been extensively studied for resistance to bacterial wilt. The identification of candidate genes in these regions, including receptor-like kinases and calcium-related signaling components, suggests potential mechanisms underlying these loci, although further validation is required.

### Limitations and next steps

Although the candidate genes identified in this study appear relevant, several limitations should be considered. First, the use of two reference genomes, while improving variant discovery, does not fully capture the structural diversity present in the panel, leaving some genomic regions inaccessible to SNP calling. Second, GWAS results are sensitive to population structure and marker density. Even if we used high-quality reference genomes and dense SNP panels with short linkage disequilibrium, these factors cannot entirely eliminate confounding effects, and the continuous, gradient-like population structure observed in our wild tomato panel may have limited the power to detect some associations or inflated the detection of small-effect loci [81]. Third, the absence of shared QTLs between temperature conditions may also partly reflect limited statistical power or environmental variability, rather than entirely distinct genetic architectures. Finally, all candidate genes identified in this study remain putative.

Therefore, future work will focus on the **functional validation of selected candidate genes** and the assessment of their stability across environments and genetic backgrounds, these validations being essential to evaluate their potential for breeding programs aimed at improving resistance to bacterial wilt under climate change. Beyond tomato, our findings have broader implications for crop improvement. As elevated temperatures are expected to alter plant-pathogen interactions across agroecosystems, identifying resistance mechanisms that are effective under heat stress is critical. In this context, the exploitation of natural diversity from wild relatives represents a promising strategy to uncover adaptive traits for future climates.

Altogether, this study demonstrates that resistance to *R. solanacearum* is **highly plastic and environmentally contingent**, and highlights the value of integrating genomic diversity, ecological context, and environmental factors to better understand plant immunity.

## Materials and methods

### Biological material

#### Tomato panel composition and R. pseudosolanacearum strain.

An initial collection of 199 tomato accessions, available in the Tomato Genetic Resource Center (University of California, Davis campus, USA), was assembled, propagated by SYNGENTA and phenotyped. Of the original collection, a panel of 189 accessions was selected and sequenced. It includes 167 accessions of *S. pimpinellifolium* species and was further enriched by adding a smaller number of accessions from related species (20 of *S. lycopersicum* var. *cerasiforme* and one of *S. cheesmaniae*) in order to capture the domestication gradient and enhance ecological and genetic diversity. Compared to cultivated tomato, which exhibits limited intraspecific DNA variation, *S. pimpinellifolium* has the lowest estimated linkage disequilibrium (LD) [46], making it well suited for GWAS. Numerous QTLs associated with pathogen resistance have been identified in this species [31–38,82], and its genome has been reported to be enriched in genes related to biotic stress responses compared to *S. lycopersicum* [62]. The cultivated ***S. lycopersicum* cv. ‘Hawaii7996’** was also included as a tolerant control for bacterial wilt [82]. *S. pimpinellifolium* species is considered one of the closest wild relatives of cultivated tomato [83], with which it can be easily crossed. It has also been proposed as the wild progenitor of *S. lycopersicum* var. *cerasiforme*, itself considered the most likely ancestor of modern large-fruited cultivated tomatoes [56]. Furthermore, climate data were retrieved from CHELSA (Climatologies at High Resolution for the Earth’s Land Surface Areas; https://chelsa-climate.org/, S10 Table). Accessions of the panel, originating from different regions of northwestern South America, likely experienced significant temperature variations, in some cases reaching up to 36,60 °C (Bio7, S10 Table), with temperatures at the collection sites ranging from -28.55 °C (BIO6, S10 Table) during the coldest months to 33.25 °C (BIO5, S10 Table) during the hottest months. BIO1 and BIO12 variables were extracted to characterize climatic conditions at each site. Passport data (S1 Table) for the accessions analyzed in this study include the accession identifier, species, country and province of origin, geographic coordinates (168 accessions) altitude (125 accessions) and BIO1/BIO2 of collection sites when available.

The whole panel was inoculated using the wild type *R. pseudosolanacearum* GMI1000 strain classified by Hong et al. [84] as a phylotype I, race 1, biovar 3 strain. It originates from South America (French Guiana) [84] and was isolated in tomato [85].

#### Plant phenotyping conditions.

For each accession, seeds were surface sterilized by immersion (5–25 seeds per 15 mL tube) in 5 mL of 20% (v/v) commercial bleach solution supplemented with 200 µL of TWEEN 20 for 10 min. Seeds were then rinsed four times with an equal volume of sterilized water and subsequently stratified in sterile water at 4°C for 48 h. Seeds were sown on rich BG solid medium [86] and allowed to germinate for 48 h at 28°C in the dark. Petri dishes were inverted during incubation to facilitate the recovery of germinated seeds. A maximum of two germinated seeds per accession were transplanted into pots (7 x 7 x 6.4 cm) filled with loam (Proveen potting soil Semi bouturage 2 with perlite, Gergy Fourniture, France). The plants were then grown for two weeks at the Toulouse Plant Microbe Phenotyping platform (INRAE-LIPME, Toulouse, France) under controlled conditions in a growth chamber [25°C day/ 24°C night, 75% RH, 12 h light, LED lights (intensity in μmol m ^− 2^ s ^− 1^ per range of wavelengths respectively at 100% Aria: 176; 400–700/ 1; 380–399/ 61; 400–499/ 20; 500–599/ 95; 600–699/ 4; 700–780/ blue, green and peak at 444, 597 and 661 nm)]. Plants were randomized at the beginning of the second week. Five days prior to inoculation, they were moved to a growth chamber in the quarantine laboratory for acclimatization under controlled conditions (28 °C day/ 27 °C night, 75% RH, 12 h light, 50 µmol m ^− 2^ s ^− 1^). Inoculations were carried out according to Morel et al. [52] by soil drenching, with 40 mL of a solution at 5 x10^7^ colony-forming unit (CFU)/mL/pot. Immediately after *R. pseudosolanacearum* inoculation, plants were transferred to growth chambers maintained at either 28 °C/27 °C (day/night) or 32 °C/31 °C (day/night). Disease progression was assessed using an established 0–4 wilting scale previously described [52], based on the proportion of wilted leaves, with the score 0 (0% of leaves wilted) and 4 (100% of leaves wilted) corresponding to healthy and dead plants, respectively. Symptoms were recorded from two to ten days after inoculation (Dai).

### Tomato genomic resource creation and analysis

#### Panel sequencing and reference genome assembly.

All panel accessions were sequenced using Illumina technology by SYNGENTA (GENEWIZ, US), targeting a raw sequencing coverage of 15x to 25x per accession (S4 and S5 Tables). The LA1246 and LA1670 accessions, representative of the two major genetic groups identified in Blanca et al [46], were selected for the *de novo* reference genome assembly. They were sequenced with a PacBio Sequel Sequencer (Pacific Biosciences, Menlo Park, CA, USA) to achieve a minimum depth of 50x. We followed a meta-assembly strategy adapted from the Rosa genome [87]. Briefly, initial contigs were generated using CANU 1.8 [88], then scaffolded with Bionano optical maps (DLE-1 enzyme, Saphyr system) *via* the hybridScaffold.pl pipeline. Detailed protocols for DNA sample preparation, sequencing and post-sequencing processing are provided in (S1 Text, S2 and S3 Tables).

#### Gene prediction and functional annotation.

For both reference genomes, protein and non-protein coding gene models were predicted using the integrative EuGene pipeline (release 1.5; http://eugene.toulouse.inra.fr/Downloads/egnep-Linux-x86_64.1.5.tar.gz; [89]). Five protein databases were aligned with NCBI-BLASTx to detect translated regions. Protein coding genes were annotated, integrating data from all databases, depending on the accuracy of all sources of information. Priority was given to BLAST-P results from the “*Solanum*” database (S2 and S3 Tables).

#### Comparative genomic analyses of reference genomes.

Analyses were performed using GENESPACE version 1.2.3 [49] to investigate genome collinearity and orthologous relationships among tomato genomes. The analysis included three annotated genome assemblies: the cultivated tomato *S. lycopersicum* ‘Heinz’ reference genome (ITAG-4.1; [48]), LA1246 and LA1670 reference genomes generated in this study. Genome assemblies and their corresponding gene annotations (GFF3 format) and predicted peptide sequences (FASTA format) were used as input. Prior to synteny inference, gene annotations were processed using the GENESPACE preprocessing workflow to retain representative gene models and ensure consistent formatting across genomes. Synteny detection and block construction were performed using the GENESPACE pipeline and default parameters. Genome collinearity and structural variation were visualized using multi-genome dot plots and riparian plots generated within the GENESPACE framework (Figs 3, S2). All analyses were conducted within the R programming language environment using GENESPACE and its associated dependencies.

#### Orthogroup analysis between LA1246 and LA1670 reference genomes.

Comparative gene analyses between LA1246 and LA1670 reference genomes were performed using OrthoFinder v3.1.0 [90]. The coding sequences (CDSs) from each accession were used as datasets. Sequence alignment was performed using BLASTN with an identity threshold of 90%. The identified orthogroups were classified based on variations in the number of gene copies between the two accessions into the following classes: absent, one copy, or multiple copies in each genome (0–1, 0-n, 1–0, 1–1, 1-n, n-0, n-1, n-n) (S6 Table). Each orthogroup was annotated with Gene Ontology (GO) terms based on existing CDS annotations *via* Blast2GO, and enriched with parent terms from the Gene Ontology DAG for the Molecular Function and Biological Process categories. GO enrichment analysis by class was performed using topGO with Fisher’s exact test [91] (S7 Table).

#### Variant calling and single nucleotide polymorphisms filtering.

Variant calling was carried out using FreeBayes [92] and the two reference genomes. Single Nucleotide Polymorphism (SNP) matrices were filtered sequentially using PLINK1.07 [93] to retain high-quality, informative and independent markers for downstream GWAS (Table 1). Filter A corresponds to initial variant calls obtained from FreeBayes, while filter B was used to remove low-quality SNPs (minQ40), non-biallelic markers, or SNPs with >5% missing genotypes. SNPs with low allele frequency were excluded to ensure a sufficient representation for reliable association testing, retaining in filter C only SNPs with a minor allele frequency (MAF) ≥ 0.032 (corresponding to at least six to seven individuals) as described previously [94]. Then filter D corresponds to a linkage disequilibrium pruning (*r²* < 0.2), performed to reduce redundancy and false-positive associations by removing highly correlated SNPs. Since preliminary tests showed an overrepresentation of heterozygous SNPs at extreme GWAS p-values, they were excluded with filter E to prevent spurious associations, ensuring a balanced representation of allelic states. After filtering, 3,728,946 SNPs (LA1670) and 3,464,481 SNPs (LA1246) were retained for GWAS (Table 1).

#### Kinship estimation and population structure.

The kinship matrix was computed using the Balding-Nichols method [95] implemented in Efficient Mixed-Model Association eXpedited (EMMAX; [96]) on SNP matrices filtered for this purpose (Table 1, filters A-D). Population structure was analysed using PCA with gdsfmt [97] R package and its snpgdsPCA() function. Results are available in (S9 Fig). Additionally, hierarchical clustering was performed using the hclust function in R on kinship-derived distance matrices for both reference genomes, identifying K = 5 distinct clusters (Figs 1A and 1D, S10A). The choice of K was based on preliminary analysis that revealed over-fragmentation with alternative K values that could separate biologically coherent groups. To assess genetic differentiation among clusters, the fixation index (F ST; [98]) was calculated in 10 kilobase (kb) windows using vcftools on the GWAS SNP matrix (Table 1, filters A-C + E; S10B Fig). Using passport data, the geographical distribution of the panel was plotted on a map of northwestern South America with the worldmap*()* function from the rnaturalearth [47] R package. Basemap data (country boundaries) were obtained from Natural Earth (public domain): https://www.naturalearthdata.com/downloads/. Terms of use: https://www.naturalearthdata.com/about/terms-of-use/.

#### Linkage disequilibrium analysis.

The LD extent (*r*^2^ measure) was investigated in two complementary approaches. First, LD half-decay was computed in a three-step procedure: GWAS matrices were sub-sampled (10% of markers per chromosome), *r*^2^ was calculated for SNP pairs from 1 bp to 1 Mbp in distance and then the mean LD was summarized within 1 kb windows. Then, to visualize chromosome-wide LD patterns, a kinship-corrected LD was computed as described in [99] with ***LDcorSV*** [100] R package using a two-step procedure. For each chromosome, 1,000 SNPs were randomly sampled from GWAS matrices, then a kinship-corrected *r*^2^ was calculated for all pairwise comparisons, providing a corrected view of LD along chromosomes (S5 Fig).

### Natural variation of QDR in the tomato panel

#### Experimental design.

To ensure balanced genotype representation and enable variance partitioning of genetic and environmental effects, phenotyping was performed using a randomized complete block design (RCBD). Five independent blocks of 450 plants were established, each comprising two technical replicates. Each replicate consisted of nine trays of 25 plants (225 plants per replicate). Within each replicate, the 198 panel accessions were randomly distributed in the nine trays with one plant per accession per replicate. The tolerant control accession ‘Hawaii 7996’ [82] was included three times per tray at fixed positions (top-left, center, bottom-right), resulting in 27 control plants per replicate.

#### Phenotypic modelization and broad-sense heritability estimation over the infection kinetic.

For each temperature x Dai combination hereafter referred to as “condition”, phenotypic values were adjusted using model **(1)**


$$\begin{array}{c} Y_{ibr}=\mu +G_{i}+B_{b}+I_{ib}+\varepsilon_{ibr} \end{array}$$ (1)

where *Y*_*ibr*_ corresponds to the phenotype of the *i*^*th*^ genotype in the *r*^*th*^ technical replicate of the *b*^*th*^ block, *μ* is the intercept, $G_{i}\sim N(\text{0},\sigma_{G}^{\text{2}})$ is the random genetic effect, $B_{b}\sim N(\text{0},\sigma_{B}^{\text{2}})$ is the random block effect, $I_{ib}\sim N(\text{0},\sigma_{I}^{\text{2}})$ is the random block x genotype interaction effect and $\varepsilon_{ibr}\sim N(\text{0},\sigma_{\epsilon }^{\text{2}})$ is the residual error. Random effects were assumed independent and normally distributed. Models were fitted by restricted maximum likelihood (REML) as implemented in the **lme4** [101] R package.

Broad-sense heritability was estimated applying the following formula **(2)** adapted from [102]:


$$\begin{array}{c} H^{\text{2}}=\frac{\sigma_{G}^{\text{2}}}{\sigma_{G}^{\text{2}}\;+\;\frac{\sigma_{B}^{\text{2}}}{n_{B}}\;+\;\frac{\sigma_{I}^{\text{2}}}{n_{I}}\;+\;\frac{\sigma_{\varepsilon }^{\text{2}}}{n_{B}n_{R}}} \end{array}$$ (2)

where *n_B_* is the number of blocks and *n_R_* the number of technical replicates.

Conditions with *H*^*2*^ > 0.1 were retained for subsequent analyses (S11 Fig), to ensure that phenotypes values included sufficient genetic signal for association mapping. At 28 °C, 7 and 10 Dai conditions were kept whereas the 4, 5, 6, 7 and 10 Dai ones were selected at 32 °C. Earlier time points were excluded due to limited disease symptom development and consequently low genetic variance.

For the retained conditions, accession-specific adjusted means (least-squares means; LS-means) were estimated using the lmerTest package in R [103] and used as phenotypic inputs for GWAS. For the models, fixed effects were tested by ANOVA, and random effects by likelihood ratio tests using the **lrtest()** function (Table 2). As 10 Dai was the time point at which the *H²* was the highest (Table 2, S11 Fig), we selected this time point to calculate the cross-temperature genetic correlations between accessions using Pearson’s correlation in order to assess the consistency of their responses across temperatures. The reaction norms were also calculated at that time point between the two temperatures on the five blocks to mitigate the potential delay in kinetics between individual blocks (S7 Fig). The three groups of phenotypic profiles observed across temperatures within the panel are presented in (Fig 2B).

#### GWAS and QTL identification.

GWAS was performed separately for each selected condition using model **(3)** described in [102] accounting for both population structure and relatedness:


$$\begin{array}{c} Y=S_{\tau }+X_{\beta }+K_{g}+\varepsilon \end{array}$$ (3)

where *Y* is the phenotype vector; *S* is the SNP incidence matrix (-1,0,1 coding); *τ* is the fixed SNP effect; *X* is the structure matrix (corresponding to the first three principal component (PC) axes); *β* is the fixed structure effect; *K* is the kinship matrix; $g\sim N\left(\text{0},K\sigma_{g}^{\text{2}}\right)$ is the random genotypic effect vector and $\varepsilon \sim N\left(\text{0},\sigma_{\varepsilon }^{\text{2}}\right)$ is the residual error. The number of PCs was selected based on the part of the variance explained for both reference genomes (S9 Fig), with the first three PCs capturing 13.8% and 11.42% of the total variance for LA1670 and LA1246, respectively.

Model (3) was fitted using EMMAX [96], applying the variance approximation [96] to cope with the large number of markers while maintaining appropriate control of type I error. Analyses were performed with the **rrBLUP** package [104].

To control False Positive Error Rate and refine QTL delineation, a local score approach was applied to SNP-level p-values [105]. This procedure aggregates statistical evidence across physically adjacent markers, allowing the identification of significant genomic intervals rather than single SNPs, thereby helping their biological interpretability. According to Bonhomme et al. [105] recommendations, the tuning parameter ξ, which controls the balance between sensitivity and specificity, was set to 3. Significant intervals were defined as putative QTLs. QTLs detected at consecutive time points and overlapping physically were merged to define consistent loci involved across infection kinetics. Underlying gene positions were retrieved from the reference genome annotations. Candidates were defined as “strict” for genes overlapping QTL intervals extended by +/-1000 bp to account for the promoter regions. Alternatively, when QTLs spanned regions outside annotated genes, the closest downstream and upstream genes were selected and defined as “intergenic candidates”.

#### Candidate genes relevance.

Because gene coordinates differed between the two reference genomes, candidate genes were reciprocally compared to assess their homology. Coding sequences were aligned against the alternate reference genome using BLASTN. Genes were considered homologous when they (i) mapped to the same chromosome; (ii) showed BLAST-N *E*-values close to zero, (iii) shared the same functional annotation; and (iv) displayed conserved genomic environments, defined by similar upstream and downstream genes.

To assess the functional relevancy of candidate genes underlying QTLs*,* Gene Ontologies annotations and predicted biological functions were investigated. Coding sequences were extracted and queried by BLASTN via the Sol Genomics network platform (https://solgenomics.net/tools/blast/) against the tomato cDNA dataset from the International Tomato Genome Sequencing Project (ITAG 4.0, [48]). Orthologs in the *A. thaliana* genome were identified using the BLASTX tool on The Arabidopsis Information Resource website (https://www.arabidopsis.org/Blast/; TAIR), selecting hits with the lowest *E*-values. Spatiotemporal expression profiles were explored in *A. thaliana* and tomato using the ePlant tools available on the Bio-Analytic Resource for Plant Biology website (https://bar.utoronto.ca).

To determine whether detected QTLs were shared or reference genome-specific and to assess potential structural variation, surrounding genomic environments, starting 1 kb upstream the start codon of the candidate genes were compared between reference genomes. Sequences, considering gene models (exons, 5’ UTR, 3’ UTR) for candidate genes, extracted from annotations of both reference genomes, were aligned using MultiAlin [106] after standardization of the start codon positions of candidate genes to zero with a custom script. Thirteen kb windows were visualized in Integrative Genomics Viewer [107]. Finally, SNP positions and corresponding p-values were added to the custom alignment script to assess SNP coverage equivalence, exon localization of top SNPs, and peak concordance between reference genomes.

QTLs were named according to the chromosome number, the sequential order along the chromosome from the proximal end and the presence of a suffix that indicates whether the QTL was detected in both reference genomes (‘BOTH’) or was specific to one of them (pedigree indicated). ‘IG’ prefixes indicate QTLs classified as intergenic. A summary of all identified QTLs is provided in (Table 3), with detailed information available in (S9 Table). Comprehensive information on the top SNPs identified on the LA1670 and LA1246 reference genomes is provided in S11 and S12 Tables, respectively. Additionally, a dataset available via a DOI given in the data availability statement section enables visualization of the allelic effects associated with each SNP.

## Supporting information

S1 Text Supporting methods. (DOCX)

S1 Table Passport data for the accessions analyzed in this study. Accession identifier, species, country and province of origin, geographic coordinates (latitude and longitude) and altitude at collection sites were retrieved when available. CHELSA (https://chelsaclimate.org/) BIO1 and BIO12 values were extracted for each accession, when available, and used to characterize climatic conditions at each collection site. BIO1 (in °C) corresponds to mean annual temperature over the 1981–2010 period calculated as the average of mean monthly temperatures over the year. BIO12 (in mm) corresponds to the sum of monthly precipitation totals across the year during the 1981–2010 period. na: not available.
(XLSX)

S2 Table *Solanum pimpinellifolium* reference accession LA1670 PacBio sequencing and analysis information. (XLSX)

S3 Table *Solanum pimpinellifolium* reference accession LA1246 PacBio sequencing and analysis information. (XLSX)

S4 Table Number of polymorphisms identified using LA1246 and mean sequencing depth per accessions. (XLSX)

S5 Table Number of polymorphisms identified using LA1670 and mean sequencing depth per accessions. (XLSX)

S6 Table Classification between LA1246 and LA1670 reference genomes of orthogroups by copy number and functional annotation. Orthogroups were classified into eight categories based on gene copy number variation between reference accessions LA1246 and LA1670: 0–1 (absent/one copy), 0-n (absent/multiple copies), 1–0 (one copy/absent), 1–1 (one copy/one copy), 1-n (one copy/multiple copies), n-0 (multiple copies/absent), n-1 (multiple copies/one copy), and n-n (multiple copies/multiple copies). Each orthogroup was annotated with Gene Ontology (GO) terms, categorized by biological process or molecular function. Comparative gene analyses between LA1246 and LA1670 reference genomes were performed using OrthoFinder v3.1.0 [90].
(XLSX)

S7 Table Gene Ontology (GO) enrichment analysis by orthogroup class. GO enrichment was performed for each genome and orthogroup class identified in S6 Table, using topGO with Fisher’s exact test [91].
(XLSX)

S8 Table Half-decay in base pairs for all chromosomes in both reference genomes. (XLSX)

S9 Table Detailed overview of the QTLs and corresponding candidate genes identified in this study. QTL name, the temperature condition (Tmp, °C) at which the QTL was detected, and the reference genome(s) in which the QTL was identified are indicated. An asterisk (*) indicates that the QTL underlying the candidate gene was detected with one reference genome but did not reach the detection p-value threshold with the other reference genome, although an association peak was observed (S8A-S8F Fig). The best *S. lycopersicum* homologous gene and corresponding annotation based on the ‘Heinz’ reference genome (ITAG4.0, 48), as well as a summary of known gene expression patterns (organs, cell types, and experimental conditions) retrieved from the ePlant tomato visualization tool (https://bar.utoronto.ca/eplant_tomato/) are provided. When available, the corresponding *A. thaliana* orthologous gene and annotation are reported. (?) indicates genes with low-confidence BLAST results. A summary of known gene expression patterns in *A. thaliana* (organs, cell types, and experimental conditions) retrieved from the ePlant visualization tool (https://bar.utoronto.ca/eplant/) is also given. Additional information includes the day after inoculation (Dai) at which the QTL was detected, gene IDs located within the identified QTL regions, the corresponding significant genomic regions (SigZones), and the top SNP names characterizing each QTL in both reference genomes (LA1246 and LA1670). na: not available.
(XLSX)

S10 Table Climatologies at High Resolution for the Earth’s Land Surface Areas (CHELSA; https://chelsa-climate.org/) data were retrieved, when available, for each accession. Descriptions of all parameters are available on the website.
(XLSX)

S11 Table Top SNPs identified along the infection kinetics by GWAS with the LA1670 reference genome. For each time point of the infection kinetics, the most significant SNPs for each QTL detected are reported, together with their minor allele frequency (MAF), the corresponding local score peak value, SNP association significance [−log_10_ (p-value)] and percentage of phenotypic variance explained (PVE).
(XLSX)

S12 Table Top SNPs identified along the infection kinetics by GWAS with the LA1246 reference genome. For each time point of the infection kinetics, the most significant SNPs for each QTL detected are reported, together with their minor allele frequency (MAF), the corresponding local score peak value, SNP association significance [-log_10_ (p-value)] and percentage of phenotypic variance explained (PVE).
(XLSX)

S1 Fig Average climate data recorded monthly between 1990 and 2019, relative to the GPS coordinates of LA1246 (A: Loja; Ecuador; 3,99S 79,36W; elevation 1259m, Köppen climate classification: Cfb) and LA1670 (B: Sama Grande; Tacna; Peru; 17,833S 70,51W; elevation 452m; Köppen climate classification: Bwh). The red line corresponds to the average temperature recorded while the average amount of precipitation is represented by blue histograms. (https://climatecharts.net/; [48]).
(TIF)

S2 Fig Global overwiev of collinearity between the reference genomes (LA1246 and LA1670) used in this study and the commonly used refernce genome ‘Heinz’. Comparisons were performed between the *de novo* assembled genome of the ‘Heinz’ reference (ITAG4.1, [50]) and our two wild accessions reference genomes generated in this study, using GENESPACE for analyses and vizualization [49].
(TIF)

S3 Fig Gene coverage by chromosome for each reference genome. Bar height indicates the mean number of SNPs per gene for each chromosome. The red line indicates the fraction of the gene containing at least one SNP. **A**: LA1246, **B**: LA1670.
(TIF)

S4 Fig Mean annual surface air temperature (A) and mean annual precipitation (B) at the sampling site, when available, during the 1981–2010 period reported on PCA results obtained with the LA1670 reference genome. Species distribution reported on PCA results obtained with the LA1670 (**C**) and the LA1246 (**D**) reference genomes.
(TIF)

S5 Fig Evolution of linkage disequilibrium (LD; *r*^*2*^) along chromosomes for both reference genomes. **A**: LA1246; **B**: LA1670. X-axis: positions on chromosomes are indicated in base pairs. Order of magnitude of half-LD is a few kb, except for chromosomes 5, 6 and 8.
(TIF)

S6 Fig LD (*r*^*2*^) between pairs of SNPs for chromosome 1 (A and B) and 8 (C and D). Raw LD is represented on the left (**A** and **C**) and LD corrected with kinship on the right (**B** and **D**). Small blocks of SNPs in LD appear on chromosome 1. However, large blocks appear on chromosome 8, which is consistent with previous results on the LD half-decay.
(TIF)

S7 Fig Effect of a 4 °C temperature elevation on plant response to *R. pseudosolanacearum* GMI1000 across the accession panel at 10 days after inoculation (Dai). Reaction norms showing the disease index (Di) at 10 Dai for each accession following inoculation at 28 °C and 32 °C. Accession-specific LS-means estimated from phenotypic values were used at each temperature. Four response profiles were defined according to the difference in disease index between 32 °C and 28 °C at 10 Dai (ΔDi = 32 °C-28 °C): (1) more susceptible at 32°C (ΔDi > 0.5); (2) less susceptible at 32°C (ΔDi < -0.5); (3) similar response at both temperatures (−0.5 < ΔDi < 0.5); and (4) symptomless accessions (−0.5 < ΔDi < 0.5 and Di 28 °C < 0.2 and DI 32 °C < 0.2), representing a subset of profile (3).
(TIF)

S8 Fig Manhattan plots, their related local score and QQ-plots. For each panel (**A-F**), data on the left correspond to results obtained with the LA1246 reference genome while data on the right correspond to LA1670. Manhattan plots obtained with the local score method (ξ = 3) are shown at the top of each panel. Red and blue lines represent the maximal and the minimal significance thresholds computed per chromosome with autocorrelation between SNPs. The Manhattan plots (with p-values; the dotted line represents the Bonferroni threshold) are displayed in the middle of each panel, and the corresponding QQ plots (with p-values) are displayed at the bottom. Each panel corresponds to a temperature condition and a Dai: **A**: 28 °C-Dai7; **B**: 28 °C-Dai10; **C**: 32 °C-Dai4; **D**: 32 °C-Dai5; **E**: 32 °C-Dai6; **F**: 32 °C-Dai7. Manhattan plots obtained with local score method at 32 °C and 10 Dai are shown in Fig 4.
(TIF)

S9 Fig Part of variance explained by PCA axes for both reference genomes. **A**: LA1246. **B**: LA1670. A slight decrease is observed when the number of axes exceeds three.
(TIF)

S10 Fig Hierarchical clustering of the panel and fixation index (F ST) within identified clusters based on the LA1670 reference genome Kinship. **A**: Kinship matrix and representation of population structure: individuals were ordered following their clustering based on PCA axes to allow blocks corresponding to population structure to appear. **B**: F ST was computed using **vcftools** (using a window size of 10 kb on the GWAS SNP matrix). Then the weighted means (by the number of markers within these windows) were computed between all combinations of the two clusters. Results are consistent between the two figures: high kinship coefficients between individuals of two different clusters lead to small F-ST values, indicating that clusters have similar genetic diversity (for example clusters 4–5).
(TIF)

S11 Fig Heritability (*H*^*2*^) calculated per day after infection for experiments performed at 28 °C and 32 °C. Blue bars indicate heritability for the 28 °C experiment and orange bars for the 32 °C one. (TIF)
