# Supplementary material for: New biologically relevant resistance to bacterial wilt in heat-stressed tomato revealed by two-reference Genome Wide Association
Source: PLoS Genet. 2026 Aug 13;22(8):e1011820. doi: 10.1371/journal.pgen.1011820 (PMC13492982; doi:10.1371/journal.pgen.1011820)
Supplement: Supplementary file 2 [file pgen.1011820.s001.docx]

**Supporting methods**

***DNA sequencing protocol***

To produce high quality DNA suitable for sequencing, between two and three young leaves were sampled from four-week-old plants (developmental stage: three to four developed leaves). High molecular weight genomic DNA was extracted following an adapted version of the protocol used in (1). The sequencing of the 189 accessions was performed by SYNGENTA (GENEWIZ, US) using Illumina chemistry. Prior to SNP calling and to increase accuracy, two accessions (LA1246 and LA1670), which were representative of the two previously identified genetic groups (2), were selected to assemble new reference genomes. To fulfill this purpose they were sequenced with a PacBio Sequel Sequencer (Pacific Biosciences, Menlo Park, CA, USA). The SMRTBell library was prepared using an “SMRTbell Express Template preparation kit” protocol. Genomic DNA (5 µg) was sheared with the 75 kbp program using a Diagenode Megaruptor (Diagenode) generating DNA fragments of approximately 25 kbp. A Femto Pulse array (Agilent Technologies, Santa Clara, CA, USA) was used to assess the fragment size distribution. Sheared genomic DNA was carried into the first enzymatic reaction to repair any damage that may be present on the DNA backbone. An A-tailing reaction, followed by the overhang adapter ligation were conducted to generate the SMRTBell template. After a 0.6 X AMPure PB Beads purification, the samples were size selected using the BluePippin (Sage Science, Beverly, MA, USA) in order to recover all the material above 20 kbp. The eluted DNA was then purified with 1 X AMPure PB Beads to obtain the final libraries of around 25 kbp. The SMRTBell libraries were quality inspected and quantified on a Femto Pulse array and a Qubit fluorimeter with Qubit dsDNA HS reagent Assay kit (Life Technologies, Thermo Fisher Scientific Inc., Waltham, MA, USA). A ready-to-sequence SMRTBell Polymerase Complex was created using a Binding Kit 2.1 (Pacific Biosciences) and the primer V4, the diffusion loading protocol was used according to the manufacturer’s instructions. The PacBio Sequel instrument was programmed to sequence the samples on PacBio SMRT cells v2.0 (Pacific Biosciences) acquiring one movie of 600 min per SMRT cell.

## *Genome assembly*

A meta-assembly strategy similar to the one used to assemble the Rosa genome (3) was applied. The Tables ‘Table-AssemblyProcessSummary-Spimpi-LA1246-11TEP’ and ‘Table-AssemblyProcessSummary-Spimpi-LA1670-12TEP’ provide details of the processing steps including data, software and the evolution of the assembly metrics. A first primary assembly was performed with CANU 1.8 (4) using one non-default parameter: corOutCoverage=200. Corrected reads generated by CANU were used as the input for SMARTdenovo (Jue Ruan, <https://github.com/ruanjue/smartdenovo>) and wtdbg2.0 software (5). The first wtdbg2.0 assembly was performed using corrected reads only, the second using both corrected reads and raw reads. The sequences of these four primary assemblies were transformed into pseudo long reads of 100 kb with an overlap of 50 kb. Then, the pseudo long reads longer than 15 kb were assembled with CANU 1.8 (–trim-assemble –pacbio-corrected).

The contigs obtained by the meta-assembly process were scaffolded using Bionano optical maps (DLE-1 enzyme, Saphyr system, hybridScaffold.pl software). The pseudo-chromosomes were obtained with ALLMAPS (6) by scaffolding the result of the hybridScaffolding.pl software using the genome S_lycopersicum_chromosomes.3.00 as a reference (the overlaps detected by "minimap2 –x asm5” were transformed into pseudo-genetic markers (~1 marker per 30 kb). Artifactual "negative gaps" (i.e. the same sequence fragment duplicated upstream and downstream gaps) generated by hybridScaffold.pl were identified and removed as well as spurious contigs (i.e. short contigs included in longer contigs; overlaps detected by minimap2 –x asm5 (7). Two iterations of polishing with arrow were performed on the raw genome sequences (including mitochondrial and chloroplast genomes, see below).

## *Assembly of mitochondrial and chloroplast genomes*

Various attempts using different software and strategies were performed in order to assemble organelle genomes (i) in a single sequence contig and (ii) with overlapping ends (allowing unambiguous circularisation). No protocol was found to resolve the two mitochondrial genomes. The mitochondrial genome of *S. lycopersicum* LA1421 (accession NC_035963.1, ‘Organellar genome analysis reveals endosymbiotic gene transfers in tomato’ (8)) was found to contain two duplications of 26813 and 46437 nucleotides. The mitogenomes of the two *S. pimpinellifolium* share a very high similarity level with the NC_035963.1 but we were unable to find in our data a raw read spanning the repeated region likely to be larger in LA1246/LA1670 compared to LA1421. Based on our data, our hypothesis is that in LA1246 and LA1670 the repeated structure spans more than 60 kb. Due to the very high similarity with LA1421, the sequence NC_035963.1 was used as the raw mitogenome of both LA1246 and LA1670 genome assemblies (despite having a different structure) in order to avoid incorrect alignments of mitochondrial reads on the nuclear genome. LA1246 and LA1670 chloroplast genomes were assembled with the protocol: trimmed reads generated by CANU 1.8 (see section and Table corresponding to genome assembly) longer than 40 kb were extracted (50 kb for LA1246) and assembled with CANU 1.8 (genomeSize=900m –pacbio-corrected –trim-assemble). Then, contigs corresponding to the chloroplasts were identified (tagged with suggestCircular = yes by CANU) and circularized (overlaps detected by BLASTN) to generate the raw chloroplast genome sequences.

All four raw genomes were polished with arrow in a process including nuclear and organelle genomes.

## *Prediction of gene models*

Protein and non-protein coding gene models were predicted using the integrative EuGene pipeline release 1.5 [(9) http://eugene.toulouse.inra.fr/Downloads/egnep-Linux-x86_64.1.5.tar.gz]. Five protein databases were aligned with NCBI-BLASTX to contribute to detection of translated regions: (i) *A. thaliana* TAIR10 proteome; (ii) Swiss-Prot - October 2016; (iii) a plant subset of Uniprot proteins – October 2016; (iv) the proteome of *Brachypodium distachyon* release 192 and (v) *Solanum lycopersicum* ITAG3.2 proteome. Proteins similar to REPBASE (20.05) were removed from the five datasets to avoid the integration of TE-related proteins in the training steps. The cleaned datasets were aligned with NCBI-BLASTX on the genomes. Chained alignments spanning less than 80 % of the length of the database protein were removed. 96 public Illumina datasets were downloaded from the SRA database (20190117; keyword *S. pimpinellifolium*; read length >= 100nt) and assembled using velvet assembler (10) in order to build a set of *S. pimpinellifolium* transcribed sequences. This set of 2933786 sequences (N50=1181nt; 2.7Gb in total) was used as transcriptional evidence by the EuGene pipeline. Spliced alignments spanning at least 30 % of the transcript length at a minimum of 97 % identity were retained. The gene modelling algorithm used the standard EuGene 4.2a parameters, except that the non canonical GC (donor) site was allowed. Regions spanned either a protein hit or by transcript alignments were preserved from the repeat masking process integrated in the pipeline. After removal of redundant ncRNA predictions: 44,253 protein-coding genes plus 2,766 non protein-coding genes were retained for LA1246 and 44,199 protein-coding genes plus 1,802 non protein-coding genes for LA1670. The BUSCO v 3.0.2 (embryophyta_odb9 reference set, 1440 BUSCO groups) was run on the set of predicted proteins. 96.9% of complete gene models plus 1.7% fragmented gene models were identified from LA1246 inferred proteins. 97.1% of complete gene models plus 1.5% fragmented gene models were identified from the predicted LA1670 proteome.

## *Functional annotation of protein-coding genes*

Protein coding genes were annotated by integrating five sources of information. Results were successively integrated depending on the expected accuracy of the source of information. Priority was successively given to: (i) a BLASTp search of reciprocal best hits with 1,354 "solanum" proteins tagged as "reviewed" in the Uniprot database (90% span, 80% identity) as of February 2019 (11); (ii) EC numbers assigned by using the protocol ((12), E2P2 v3.1) with BLAST e-value cutoff lowered to 1.e^-5^ and pathway-prediction-score set to 0.3 in pathway-tools; (iii) the transcription factors and kinases identified by ITAK release 1.7 (13); (iv) the transcription factors identified by PlantTFCat (14); (v) the Interpro (release 72.0) search (15). The EC numbers were tested against the ENZYME (16) database (release February 2019), updated when out-of-date and then used to get the description of the enzymes. Finally, the protein annotations were validated by the tbl2asn software (https://www.ncbi.nlm.nih.gov/genbank/tbl2asn2, February 2019). GO terms were assigned using the BLAST2GO pro software integrating BLASTp similarities with Genbank NR ((17); 2017-12-08, e-value < 1e-5, 20 best-scoring hits) and Interpro release 72 results.

## *References*

1.	Mayjonade B, Gouzy J, Donnadieu C, Pouilly N, Marande W, Callot C, et al. Extraction of high-molecular-weight genomic DNA for long-read sequencing of single molecules. BioTechniques. 2016 Oct;61(4):203–5.

2.	Blanca J, Montero-Pau J, Sauvage C, Bauchet G, Illa E, Díez MJ, et al. Genomic variation in tomato, from wild ancestors to contemporary breeding accessions. BMC Genomics. 2015 Dec;16(1):257.

3.	Raymond O, Gouzy J, Just J, Badouin H, Verdenaud M, Lemainque A, et al. The Rosa genome provides new insights into the domestication of modern roses. Nat Genet. 2018 Jun;50(6):772–7.

4.	Koren S, Walenz BP, Berlin K, Miller JR, Bergman NH, Phillippy AM. Canu: scalable and accurate long-read assembly via adaptive k-mer weighting and repeat separation. Genome Res. 2017 Jan 5;27(5):722–36.

5.	Ruan J, Li H. Fast and accurate long-read assembly with wtdbg2. Nat Methods. 2020 Feb;17(2):155–8.

6.	Tang H, Zhang X, Miao C, Zhang J, Ming R, Schnable JC, et al. ALLMAPS: robust scaffold ordering based on multiple maps. Genome Biol. 2015 Jan 13;16(1):3.

7.	Li H. Minimap2: pairwise alignment for nucleotide sequences. Bioinformatics. 2018 Sep 15;34(18):3094–100.

8.	Kim HT, Lee JM. Organellar genome analysis reveals endosymbiotic gene transfers in tomato. PLOS ONE. 2018 Sep 5;13(9):e0202279.

9.	Sallet E, Gouzy J, Schiex T. EuGene: An Automated Integrative Gene Finder for Eukaryotes and Prokaryotes. In Kollmar, M. (eds) Gene Prediction. Methods in Molecular Biology, Humana, New York. 2019 Apr ; 1962 :97-120.

10.	Zerbino DR, Birney E. Velvet: Algorithms for de novo short read assembly using de Bruijn graphs. Genome Research. 2008 Jan 5;18(5):821–9.

11.	The UniProt Consortium. Update on activities at the Universal Protein Resource (UniProt) in 2013. Nucleic Acids Research. 2013 Jan 1;41(D1):D43–7.

12.	Schläpfer P, Zhang P, Wang C, Kim T, Banf M, Chae L, et al. Genome-Wide Prediction of Metabolic Enzymes, Pathways, and Gene Clusters in Plants. Plant Physiology. 2017 Apr 1;173(4):2041–59.

13.	Zheng Y, Jiao C, Sun H, Rosli HG, Pombo MA, Zhang P, et al. iTAK: A Program for Genome-wide Prediction and Classification of Plant Transcription Factors, Transcriptional Regulators, and Protein Kinases. Molecular Plant. 2016 Dec;9(12):1667–70.

14.	Dai X, Sinharoy S, Udvardi M, Zhao PX. PlantTFcat: an online plant transcription factor and transcriptional regulator categorization and analysis tool. BMC Bioinformatics. 2013 Nov 12;14(1):321.

15.	Finn RD, Attwood TK, Babbitt PC, Bateman A, Bork P, Bridge AJ, et al. InterPro in 2017—beyond protein family and domain annotations. Nucleic Acids Research. 2017 Jan 4;45(D1):D190–9.

16.	Bairoch A. The ENZYME database in 2000. Nucleic Acids Research. 2000 Jan 1;28(1):304–5.

17.	Benson DA, Cavanaugh M, Clark K, Karsch-Mizrachi I, Lipman DJ, Ostell J, et al. GenBank. Nucleic Acids Research. 2013 Jan 1;41(D1):D36–42.
