# Supplementary material for: New biologically relevant resistance to bacterial wilt in heat-stressed tomato revealed by two-reference Genome Wide Association
Source: PLoS Genet. 2026 Aug 13;22(8):e1011820. doi: 10.1371/journal.pgen.1011820 (PMC13492982; doi:10.1371/journal.pgen.1011820)
Supplement: Supplementary file 3 [file pgen.1011820.s025.docx]

The article « New resistance to bacterial wilt in heat-stressed tomato is revealed by two-reference Genome Wide Association » by Belny et al. is suitable for publication in Plos Genetics with major revisions.

The study investigated temperature-dependent quantitative disease resistance (QDR) to bacterial wilt caused by Ralstonia solanacearum in tomato relatives. A unique panel of 189 accessions was composed, re-sequenced and phenoytped for their tolerance to the pathogen at two temperatures. An original GWAs approach, based on the use of two reference genomes for the set up of the SNP matrix was conducted to uncover the genetic basis of the resistance. Several candidates genes (CG) could be identified. The authors demontrasted that those CG, mostly already known to be involved in plant response immunity, followed a temporal dynamics of activation during infection progress. Surprisingly, no candidate genes were common between the two phenotyping temperatures (28∘C and 32∘C). This highlights that the genetic mechanisms of QDR are distinct and temperature-specific. Two of the newly identified CG are closely linked to the genomic positions of the two major QTLs already described for bacterial wilt QDR. Some other CG are also relevant given the available literature. The authors emphasize that the characterized genetic resource is valuable for identifying new sources of thermostable resistance. However, it appears the results actually suggest the inverse: that the genetic mechanisms underlying resistance are different across the various temperatures tested. This challenges the conclusion of 'thermostable' resistance." Overall, the study's results suggest that the wild relatives of tomato harbor a diverse and temperature-dependent genetic arsenal against bacterial wilt, offering novel targets for breeding efforts aimed at overcoming the increased disease susceptibility faced by commercial varieties under rising temperature conditions.

Specific comments have been directly added in the pdf file using the reviewer comments mode.

In brief for the major comments :

On the analysis of the panel :

The panel is mostly composed of *S. pimpinellifolium*. The authors should detail the scientific rationale for including the others species accessions in the panel. The results (PCA, phenotyping, allelic frequencies) should also be analysed and interpret accordingly.

The discussion section could specifically highlight the genetic results (QTLs/associated variants, allele origin) in the context of the accessions' geographical origin and biome. This approach would allow the authors to directly address and test their hypothesis regarding biome-dependent resistance mechanisms.

On the reference genomes.

We encourage the authors to include a deeper comparative analysis of the genomes, focusing specifically on presence/absence of genes.

On the statistical analyses

The results of the statistical analyses performed on the phenotypic data should be more developed (ANOVA results, correlation, reaction norms …). The traits under studied should be explicitly listed. The authors could also add other estimated traits such as AUDPC or kinetics parameters

On QTL analyses

A consolidated figure representing all identified genomic regions (loci) is recommended. For each significant locus, a dedicated figure illustrating its effect (or significance) across the temporal measurement points would greatly enhance clarity.

For each detected QTL, the authors should provide essential quantitative metrics, including the substitution allelic effect, the percentage of explained phenotypic variance, and the Minor Allele Frequency (MAF) of the associated marker. This information is critical for interpreting the biological significance of the results.

The discussion section could be restructured. The functional analyses of the candidate genes could be shorten. A conceptualy figure describing the main hypothesis about the mechanisms underlying multiple stress response could be very impactful.
