# Supplementary material for: New biologically relevant resistance to bacterial wilt in heat-stressed tomato revealed by two-reference Genome Wide Association
Source: PLoS Genet. 2026 Aug 13;22(8):e1011820. doi: 10.1371/journal.pgen.1011820 (PMC13492982; doi:10.1371/journal.pgen.1011820)
Supplement: Supplementary file 4 [file pgen.1011820.s027.docx]

Dr Richard Berthomé

Senior scientist in the « Resistance Pathway Dynamics and

Adaptation to Climate Change » team

Laboratoire des Interactions Plantes-Microorganismes-Environment (LIPME)

UMR CNRS/INRA 2594/441

24 Chemin de Borde Rouge,

CS 52627, 31326 Castanet-Tolosan cedex, FRANCE.

Tel +33 (0)658285126

PLOS Genetics Editorial board

Toulouse, 5 April 2026

**Subject:** Response to reviewers’ comments ; manuscript ID PGENETICS-D-25-00847

*Dear Editor,*

*we sincerely thank you and the reviewers for your constructive and helful comments, which clearly contributed improving our manuscript. We have carefully considered all suggestions and revised the manuscript accordingly. The main comments, which summarize the major points raised by the three reviewers, are addressed in detail below and within the annotated reviewers’ reports. All major revisions in the manuscript and responses to comments have been highlighted for ease of reference.*

*We hope that the revised manuscript will meets the standards of the journal*

*Sincerely*

*Richard Berthomé*

*Corresponding author*

- It would be better to begin the abstract and the introduction by general statements on the scientific topics rather than on specificities about the model organisms.
- The lack of clarity in the experimental design, the choice of accessions, statistical analyses, and data presentation,
- Better present the QTL results, with additional figures
- Perform a more comprehensive comparison of the two reference genomes.
- The analysis of population structure needs improvements.
- Tone down claims about gene functions, the existence of thermostable resistance and the utility of the two reference genomes, highlighting that the inference about so called "thermostable resistance" should actually be the opposite.
- Discuss the findings in relationship with the accession origins,
- Discuss the study limitations.
- There are too many abbreviations, this renders the text harder to read without providing benefits.

*To address all of these recommendations, we have:*

1) *Restructured the Introduction, adding clarifications as requested (contextual background, rationale for choices, and additional details).*
2) *Revised the Materials and Methods section to improve clarity, explaining the rationale for the selection of the panel and providing additional details on its composition. The description of the experimental design has also been revised for better presentation. We have updated the description of statistical analyses, explaining the reasoning behind their use. Additional analyses enabling comparisons of the reference genomes used in this study have been included, with the results described and discussed. Interpretations of analyses related to population structure have also been expanded.*
3) *Perfomed additionnal analyses (genomes comparisons).*
4) *Presented the results in new figures (Figures 1, 2, 5, S1 & S9 Fig), revised figures (Figures 3 and 4), new tables (Table 2, Supplementary Tables), and reformatted Tables (Table 3, S7 Table), providing the data used or generated in this study, including complete information on all top SNPs underlying the various QTLs identified with the two reference genomes (S8 & S9Tables).*
5) *Reformatted all figures (300 dpi) to meet journal standards and converted them to .tiff format.*
6) *Updated the .git repository to include climate data collected for the analyzed accessions, as well as the scripts used to generate the analyses and figures (Figure 2, Fig S9).*
7) *Give an access to additional figures (numerous…) illustrating the allelic effects of the top SNPs corresponding to the QTLs, complementing S8 and S9 Tables (link in the Data Availability Statement section).*

*In light of these additional and complementary results, the discussion section has been revised accordingly. We have reconsidered the interpretation of our findings in response to the reviewers' comments and included a section on the interpretation of our results in relation to the environmental origin of the panel accessions, on the limitations of our study and future prospects. As suggested, the Abstract has also been revised, and statements throughout the manuscript have been moderated where appropriate.*

*Below, you will find responses to specific comments and to the reviewers*

-Please include page numbers.

*Page numbers were added.*

-L87: define PAMP

*This part of the introduction was revised and shorten as suggested by R3.*

-P11-12: there are too many abbreviations and specific details, this renders the text harder to read

*The text of both pages was revised*

-L133-134; L166-167: unclear what you mean

*Clarifications have been added, and the relevant section has been revised where necessary*.

-P14: unclear if these "wild relatives" all belong to a single species? a single population?

*The composition of the panel and the rational for its selection were explained in detail and clarified in the Materials and Methods section.*

-L168: cut the sentence into two

*These elements have been updated following the revision of the Introduction.*

-L375-7: the species involved should be clear from the introduction; the text mentions 5 species but only four are listed; why including different species, especially the two with a single accession?

*Additional details have been provided in the Introduction and Materials and Methods sections.*

-L386-390: unclear, two references are said to be needed because one species from different clusters, but what about the other species? The rationale for choosing different environments for a reference is unclear.

*In line with our working hypothesis, the panel composition, including the enrichment in S. pimpinellifolium accessions and the choice of internal reference accessions, has been clarified in the Introduction and Materials and Methods sections.*

-Figure 1: unclear if these includes the five species and the correspondence with the clusters; the species delimitation could even be discussed given the lack of clear genetic subdivision; the figure and the text are unclear about how the clusters were delimited. Analyses such as STRCUTRE and Splitstree should be run. The delimitation into these 5 clusters is not convincing at all based on the PCAs.

*Figure 1 has been renumbered as Figure 2, and the species composing the panel (with the rationale for their selection clarified) are now indicated on the PCA results for each reference genome in Fig S9.*

-Figure 2: not sure this is needed as a main figure

*We agree with the editor. This illustration has been moved to the supporting information, and a new Figure 1 presenting the phenotyping results has been created.*

-L431: it does change the separation of the five so called clusters ?

*Indeed, with only minor effects on the clusters. In light of our results, the comparison of reference genomes, and our GWAS findings, these observations have been discussed.*

-L433: the term diversified is not correct here; give numbers of differences

*We agree that the term diversified was not appropriate. However, the genome comparison requested by the reviewer, which is particularly relevant, highlights differences between the two references (CNVs, presence/absence of genes, orthogroups, local rearrangements), confirming their value.*

-L439: the term "share" is not correct here, FST is a value for a pair

*This has been corrected.*

-Define all abbreviations in all figure legends

*This has been corrected accordingly.*

-L488: high, not important

*This has been corrected*

-P33: a more comprehensive analysis of structural variants and better representation are needed

*New analyses enabling a better comparison of the reference genomes have been performed, and tables / figures created for this purpose (Tables S8 & S9, Figure 2, Fig S9). Additionally, Figure 4 has been revised to provide a clearer representation of the examples presented.*

-L588: not exactly, the variation in the sample for the response to the disease was different at the two temperatures, but there may be a shared regulation that is not variable among accession

-L455-456: this is just the disease progressing, not susceptibility increasing

*Using ratios that compare accessions based on the disease index allows the identification of distinct response profiles (new Figure 1B). These profiles are supported by reaction norms at 10 Dai at both temperatures, indicating the presence of different genetic architectures underlying combined stress responses (S6 Fig). One profile corresponds to a majority of accessions that become more susceptible.*

-L603: delete "interestingly"; based on what evidence was the previous annotation?

-L608: what does "particularly expressed » means? upregulated ?

-L632 : « assumption » is not the right term here, it is more a « hypothesis »

-L634 : unclear ; what is accentuated ?

*As the discussion has been revised, this element has been clarified.*

-Do not refer to figures or Tables in the discussion, they should have explained well enough in the result section

*We have removed these references, except in the discussion section ‘Traces of local environmental adaptation in the panel,’ where the discussion is supported by a new illustration (Figure 5).*

-L637 : who is « they » ?

-L638, L674, L729: delete "interestingly";

*This has been corrected*

-L639-643: this is a bit misleading, the results do not show well defined clusters in the response to disease, it looks more like a continuous trait homogeneous, saying it has lost diversity does not make sense, especially when using a single genome as a reference anyway

*Same response as above : using ratios that compare accessions based on the disease index allows the identification of distinct response profiles (new Figure 1B, we agree, not well defined clusters…). These profiles are supported by reaction norms at 10 Dai at both temperatures, indicating the presence of different genetic architectures underlying combined stress responses (S6 Fig)*

-L650: reduction in what?

-L614, L662: your question is unclear: do you look for resistance at high temperature, or at both temperature? This is different: clarify and discuss the results given the previse question

-L664: why is geographical distance important?

-L672: what do you mean by unique here?

*We have clarified or corrected these points in the discussion section of the current manuscript.*

-L671, 713, 736, 762, 771, 781: remove bold

*This has been changed accordingly*

-The discussion should end by broadening the context to other crops

*The discussion has been fully revised, with particular attention to this point.*

**Reviewer #1**

Major comments:

1. The authors emphasize thermostable resistance, yet no QTL were common to 28 °C and 32 °C. How do they justify the term “thermostable candidate loci” (see Abstract line ~57, Author summary) As field temperatures fluctuate below 28 °C and above 32 °C, it is unclear how results restricted to condition-specific QTL translate to breeding for stable resistance. Please clarify this interpretation.

*We agree with the reviewer and have modified/expanded our interpretations. We did not identify candidate genes corresponding to stable plant defense responses, but rather candidate genes that may be involved in time- and temperature-dependent responses to R. pseudosolanacearum.*

2. Although the Results mention cases where SNPs could not be detected in LA1246, the Discussion does not explicitly address limitations. Please add a dedicated limitations paragraph.

*We have added a section discussing the limits of our study.*

3. In the Author summary, the authors state that some candidate loci are “currently undergoing functional validation.” If this is work planned by the authors, please move this point to a future directions statement in the Discussion.

*We have removed this from the Abstract and instead mention the need to validate these candidates at the end of the discussion, in the ‘Limitations and Next Steps’ section.*

4. The Introduction (lines ~152) states that optimal growth for tomato is 13–33 °C, yet 32 °C is used as the “heat stress” condition. Evidence is needed that the specific accessions studied have optimal growth below 32 °C. For example, a map of mean annual temperatures at accession origins would substantiate that 32 °C imposes stress relative to their natural ranges.

*We retrieved air temperature and precipitation data from the collection sites of the accessions for the period 1981 - 2010. These data show that the average temperatures under which the accessions grow are well below 32 °C, although some accessions, depending on their origin, may experience temperatures up to 36 °C. These data are available in S1 & S2 Tables and were used in the new Figures 3 and S9 Fig.*

5. The phenotyping protocol (Materials & Methods) describes seed sterilization and transplanting but does not state clearly how many biological replicates per accession were used.

*The experimental design have been better explained and described in the material and method section.*

6. The inoculation method references Morel et al. (49) but does not specify whether root-dip, soil-drench, or stem injection was used. Please clarify. And the 0 - 4 disease index scale is not explained. A brief description of what each score corresponds to should be added.

*We thank the reviewer for this comment and have clarified the inoculation method used (soil drenching) in the corresponding section. The 0-4 disease index scale is now explained in the current manuscript.*

7. The manuscript shows a Bonferroni line in Manhattan plots but does not explain the correction method.

*We used the local score method to detect QTLs throughout this analysis. Classic Manhattan plots and QQ-plots have been provided in the Supporting Information as part of our QC of the analyses.*

8. Significant SNPs’ p-values are not reported in supplementary tables.

*They are now given in S8 and S9 Tables.*

9. Given >3 million SNPs, Bonferroni is extremely strict (p ≈ 1.3 × 10⁻⁸). Consider also reporting FDR thresholds.

*We used the local score method to detect QTLs throughout this analysis. Classic Manhattan plots and QQ-plots have been provided in the Supporting Information as part of our QC of the analyses.*

10. MAF was set to 0.032 (≈12 minor alleles in 189 diploids). This is low and risks unstable estimates. Please show the formula cited, explain why this cutoff was chosen.

*We applied filter E in the GWAS, ensuring at least six individuals per allelic class (00, 01, 11), corresponding to a minimum MAF of 0.048.*

11. Kinship estimation used filters A+B+C+D while GWAS used A+B+C+E. Please explain the rationale for using different SNP sets for kinship vs association.

*Two different filters were applied because the requirements for kinship calculation and GWAS differ. For kinship, independent markers or markers in low linkage disequilibrium are needed to avoid overestimating relationships, which justifies filter D. For GWAS, markers in LD are useful to detect associations, so a less stringent filter is applied*

12. Three PCs were included in GWAS; please report the variance explained by these PCs.

*The variance explained by the first three axes are reported in Figure S2*

13. In the LA1246-based PCA, LA1246 and ‘Hawaii 7996’ cluster together. Is this due to introgression from S. pimpinellifolium into ‘Hawaii 7996’ or lack of sampling from S. lycopersicum? Please discuss.

*This has been discussed in the revised version of the manuscript L694 : Notably, the clustering of ‘Hawaii 7996’ with a subset of accessions including the reference LA1246 on PCA may reflect introgression from wild relatives, as previously proposed (50).*

14. Please provide a supplementary table with ancestry proportions of the five clusters, and consider comparing phenotypes across clusters to relate genetic diversity to resistance.

*We thank the reviewer for the suggestion. Population structure was assessed using Principal Component Analysis (PCA) and hierarchical clustering on kinship-derived distance matrices for both reference genomes, which identified five distinct clusters (Figure 3; S3 Fig A,B). The choice of K=5 was guided by preliminary analyses: alternative values of K led to over-fragmentation, separating groups that are biologically coherent and reducing interpretability.* *Such analyses could be considered in future work.*

Minor comments:

o “Solanaceaous” → Solanaceae.

*This has been corrected.*

o Lines 86–93 (extended PTI vs ETI zig-zag model explanation) feel redundant and distract from the main story. Consider shortening.

*We revised this section and shorten it.*

o T3E is introduced at line 113 without prior definition. Please define “type III effectors (T3E)” when first mentioned (~line 103).

*This has been corrected (lines 98 and 113)*

o Example: line 98 cites (3,2); line 108 cites (17,14). Please fix order and format.

*We thank the reviewer for this comment. We have reviewed the entire manuscript to correct this issue*.

o Figure 1 legend: clarify “A–C” should read “A & C” and “B–D” should read “B & D” to match panels. Also, each figure should indicate clearly which reference genome is used.

*Figure 1 has been updated. The new figure was revised using models A and C.*

o Figure 3: the three response groups should be more clearly distinguished; adding representative plant images would strengthen the figure.

It is not possible to provide a representative photograph for each group, as the figure shows a *comparison of adjusted mean ratings (lsmeans) for each accession of the panel, calculated from experiments conducted separately at 28°C and 32°C. The classical phenotypic responses corresponding to the disease index scale is already published with representative plant images (, a revised Figure 2 has been created.*

o Figure 5: Panel A y-axis should be labeled “local score.” Panel B legend refers to arrows that are not visible; please correct.

*Figure 5 corresponds to the new figure 4. These points were corrected.*

o Table 3: add a column with p-values for candidate SNPs/QTL.

*They are given in S8 and S9 Tables.*

o Results line 375: the panel included four species, not five (167 S. pimpinellifolium, 20 S. lycopersicum var. cerasiforme, one S. cheesmaniae, one S. lycopersicum cultivar). Please correct.

*The composition of the panel (species) and the rationale for this choice are better explained in the revised version of our manuscript.*

o Please state whether any non-inoculated wild accessions were grown at both temperatures to confirm symptoms were disease-related and not physiological stress.

*We thank the reviewer for this comment. In previous experiments, non-inoculated accessions were grown at both temperatures to confirm that symptoms were disease-related rather than due to physiological stress in infected plants. Additionally, bacterial multiplication in planta was verified. For the current experiment, given the large number of plants phenotyped, including a sufficient number of non-inoculated controls was not feasible due to space constraints. However, since inoculation with the bacterium led to systematic death of certain accessions, we were able to observe both tolerant and sensitive controls within the inoculated plants*.

o “GWA” (line 483) should be written consistently as “GWAS.”

*This has been corrected*

**Reviewer #2:**

-Not sure why the data set was reduced from 199 to 189

*The rationale for the selection of the panel is now better explained in the current version of the manuscript*

-Percentages of the altitude of origin don’t add up.

*We agree. These data, however, remain available in the passport data.*

-It is extremely unlikely to find Pimpinellifolium or Cerasiformae above 3000 m. Please check your records again.

*These data were obtained from a biological resource center. We did not perform field collection ourselves (although we would have liked to!)*

-You first report 21 candidates, later only 19 homologs?

*The effective number of QTLs and candidate genes has been verified in the revised version.*

-Can you authors explain a bit more why it is not weird that there was no overlap between QTL? Only 3 degrees difference?

*Even though we did not apply a heat shock, a constant 4 °C increase represents a significant stress. The rationale for using this magnitude is now better explained in the manuscript. Temporal and temperature-dependent differential responses are discussed in the current version of the manuscript.*

-Did the authors test whether the plants were stressed beforeand like by measuring Fv/Fm or other measures of respiratory fitness?

*We thank the reviewer for this comment. In fact, we did not do this for the current study, but it is something we plan to implement systematically in future field experiments. In previous experiments, non-inoculated accessions were grown at both temperatures to confirm that symptoms were disease-related rather than due to physiological stress in infected plants. Additionally, bacterial multiplication in planta was verified. For the current experiment, given the large number of plants phenotyped, including a sufficient number of non-inoculated controls was not feasible due to space constraints. However, since inoculation with the bacterium led to systematic death of certain accessions, we were able to observe both tolerant and sensitive controls within the inoculated plants.*

-Whereas the study is useful, claiming using two reference for different GWAS is not the same as offering alternatives for Pangenomes, the relative sections should be tuned down
-The functional claims of the GWAS candidates are solely based on Arabidopsis annotations. This should be properly placed in context without overstatement
- The discussion could be tuned down. There were no actually validated candidate genes. I also don’t think that is needed for this study, but until real validation happens… I also think the time and temperature dependent results should be discussed a bit more in light of possible false positives or why the authors think all these loci are real.

*Our interpretations have been revised and moderated in the current manuscript.*

**Reviewer #3:**

Major comments

On the analysis of the panel

The panel is mostly composed of S. pimpinellifolium. The authors should detail the scientific rationale for including the others species accessions in the panel. The results (PCA, phenotyping, allelic frequencies) should also be analysed and interpret accordingly.

*The rationale behind the selection of this panel, the experimental design, the methods, and the results from previous or new analyses have allowed us to better interpret and discuss the findings in this revised version of the manuscript.*

The discussion section could specifically highlight the genetic results (QTLs/associated variants, allele origin) in the context of the accessions' geographical origin and biome. This approach would allow the authors to directly address and test their hypothesis regarding biome-dependent resistance mechanisms.

*We thank the reviewer for this suggestion. New analyses, results obtained, and figures generated allowed us to revise the discussion to better highlight the genetic results (QTLs, associated variants, allele origin) in the context of the accessions’ geographical origin and biome.*

On the reference genomes.

We encourage the authors to include a deeper comparative analysis of the genomes, focusing specifically on presence/absence of genes.

*A deeper comparative analysis of the reference genomes focusing on synteny conservation and gene presence/absence across orthogroups was performed. This analysis led to the creation of the new* ***Figue 2, S1 Fig****,* ***S5 and S6-S11 Tables****. These results are now described and discussed in our revised manuscript as suggested by the reviewer.*

On the statistical analyses

The results of the statistical analyses performed on the phenotypic data should be more developed (ANOVA results, correlation, reaction norms …). The traits under studied should be explicitly listed. The authors could also add other estimated traits such as AUDPC or kinetics parameters

*The Materials and Methods section entitled “Phenotypic modelization and broad-sense heritability estimation over the kinetic” has been revised to clarify the statistical analyses. We now explicitly indicate the statistical methods used, as well as the R packages and functions employed to test the fixed and random effects of the models and to estimate cross-temperature genetic correlations between accessions. In addition, the description of the trait has been improved.*

*We thank the reviewer for this suggestion (AUDPC or kinetics parameters). Estimating additional traits such as AUDPC or kinetic parameters would provide complementary information on disease progression. However, the aim of the present study was to focus on disease severity at specific time points to characterize temperature-dependent responses within the panel. Therefore, additional derived traits were not included in the present study. However, we agree that such approaches are valuable. We have explored these aspects in a new set of experiments conducted under field conditions across two seasons and over three years. These analyses, including the estimation of kinetic parameters and AUDPC are being developed in a separate manuscript currently in preparation.*

On QTL analyses

**A consolidated figure representing all identified genomic regions (loci) is recommended. For each significant locus**, a dedicated figure illustrating its effect (or significance) across the temporal measurement points would greatly enhance clarity.

For each detected QTL, the authors should provide essential quantitative metrics, including the substitution allelic effect, the percentage of explained phenotypic variance, and the Minor Allele Frequency (MAF) of the associated marker. This information is critical for interpreting the biological significance of the results.

*A new Figure 2 has been added to present the results of the comparative analysis of the reference genomes. This figure illustrates the synteny relationships among the reference genomes (Figure 2 C and D, S1 Fig) as well as with the Heinz reference genome (Figure 2 A and B, S1 Fig), and indicates the positions of the QTL identified with each reference genome at the different temperatures and Dai (Figure 2 D). Box plots illustrating the effect of each top SNPs for each QTL are provided via the following DOI : https://zenodo.org/uploads/19391497  (S12 Figs)****.*** *Their quantitative metrics are reported for each reference genome in S8 and S9 Tables.*

The discussion section could be restructured. The functional analyses of the candidate genes could be shorten. A conceptualy figure describing the main hypothesis about the mechanisms underlying multiple stress response could be very impactful.

*We thank the reviewer for these valuable suggestions. The discussion has been revised to include further consideration of the use of two reference genomes, as well as the composition, origins, and characteristics of the panel in relation to the results obtained. The section on the biological relevance of candidate genes has been shortened, as suggested, while highlighting the need for functional validation of the identified candidates, as carried out in our previous studies. Although these candidates appear biologically meaningful, validation of the most promising ones is still ongoing, and it may be premature at this stage to propose a conceptual model. Such a model would require more extensive meta-analyses across similar studies, which remain limited. Nevertheless, we agree that this is an interesting direction for future work.*

**Journal Requirements:**

If the reviewer comments include a recommendation to cite specific previously published works, please review and evaluate these publications to determine whether they are relevant and should be cited. There is no requirement to cite these works unless the editor has indicated otherwise.

1) Please upload all main figures as separate Figure files in .tif or .eps format. For more information about how to convert and format your figure files please see our guidelines:
   https://journals.plos.org/plosgenetics/s/figures

.tif

2) We have noticed that you have cited Table  2 in the manuscript file but there is no corresponding table in the manuscript.  Please amend your manuscript to include this table noting that tables should not be uploaded as individual files.

3) We have noticed that you have a list of Supporting Information legends in your manuscript. However, there are no corresponding files uploaded to the submission. Please upload them as separate files with the item type 'Supporting Information'.

*I agree and apologize for this oversight. I was on medical leave at the time of the initial submission and was therefore unable to verify this. Supporting information, including S1 Text, S1 and 12 Tables, and S1 and S11 Figures, will be uploaded.”*

4) Some material included in your submission may be copyrighted. According to PLOSu2019s copyright policy, authors who use figures or other material (e.g., graphics, clipart, maps R package Sf avec l’auteur) from another author or copyright holder must demonstrate or obtain permission to publish this material under the Creative Commons Attribution 4.0 International (CC BY 4.0) License used by PLOS journals. Please closely review the details of PLOSu2019s copyright requirements here: PLOS Licenses and Copyright. If you need to request permissions from a copyright holder, you may use PLOS's Copyright Content Permission form.

Please respond directly to this email and provide any known details concerning your material's license terms and permissions required for reuse, even if you have not yet obtained copyright permissions or are unsure of your material's copyright compatibility. Once you have responded and addressed all other outstanding technical requirements, you may resubmit your manuscript within Editorial Manager. Potential Copyright Issues:

i) Figure 1. Please (a) provide a direct link to the base layer of the map (i.e., the country or region border shape) and ensure this is also included in the figure legend; and (b) provide a link to the terms of use / license information for the base layer image or shapefile. We cannot publish proprietary or copyrighted maps (e.g. Google Maps, Mapquest) and the terms of use for your map base layer must be compatible with our CC BY 4.0 license.
Note: if you created the map in a software program like R or ArcGIS, please locate and indicate the source of the basemap shapefile onto which data has been plotted.
If your map was obtained from a copyrighted source please amend the figure so that the base map used is from an openly available source. Alternatively, please provide explicit written permission from the copyright holder granting you the right to publish the material under our CC BY 4.0 license.
If you are unsure whether you can use a map or not, please do reach out and we will be able to help you. The following websites are good examples of where you can source open access or public domain maps:
* U.S. Geological Survey (USGS) - All maps are in the public domain. ([http://www.usgs.gov](http://www.usgs.gov/))
* PlaniGlobe - All maps are published under a Creative Commons license so please cite u201cPlaniGlobe, [http://www.planiglobe.com](http://www.planiglobe.com/), CC BY 2.0u201d in the image credit after the caption. (<http://www.planiglobe.com/?lang=enl>)
* Natural Earth - All maps are public domain. (<http://www.naturalearthdata.com/about/terms-of-use/>).

*Using passport data for our panel, we were able to present its geographical distribution on a map of northwestern South America. The points were plotted using the worldmap() function from the rnaturalearth R package. The reference is cited in the manuscript, and this information is provided in the Materials and Methods section.*

Massicotte P, South A rnaturalearth: World Map Data from Natural Earth. 2026. R package version 1.2.0.9000, <https://docs.ropensci.org/rnaturalearth/>.

5) Please amend your detailed Financial Disclosure statement. This is published with the article. It must therefore be completed in full sentences and contain the exact wording you wish to be published.
1) State the initials, alongside each funding source, of each author to receive each grant. For example: "This work was supported by the National Institutes of Health (####### to AM; ###### to CJ) and the National Science Foundation (###### to AM)."
2) State what role the funders took in the study. If the funders had no role in your study, please state: "The funders had no role in study design, data collection and analysis, decision to publish, or preparation of the manuscript."
3) If any authors received a salary from any of your funders, please state which authors and which funders..
If you did not receive any funding for this study, please simply state: u201cThe authors received no specific funding for this work.u201d

6) Kindly revise your competing statement in the online submission form to align with the journal's style guidelines: 'The authors declare that there are no competing interests.'

*This has been done in the current version of the manuscript.*
