# Supplementary material for: New biologically relevant resistance to bacterial wilt in heat-stressed tomato revealed by two-reference Genome Wide Association
Source: PLoS Genet. 2026 Aug 13;22(8):e1011820. doi: 10.1371/journal.pgen.1011820 (PMC13492982; doi:10.1371/journal.pgen.1011820)
Supplement: Supplementary file 5 [file pgen.1011820.s028.docx]

Dr Richard Berthomé

Senior scientist in the « Resistance Pathway Dynamics and

Adaptation to Climate Change » team

Laboratoire des Interactions Plantes-Microorganismes-Environment (LIPME)

UMR CNRS/INRA 2594/441

24 Chemin de Borde Rouge,

CS 52627, 31326 Castanet-Tolosan cedex, FRANCE.

Tel +33 (0)658285126

PLOS Genetics Editorial board

Toulouse, 11 june 2026

**Subject:** Response to reviewers’ comments ; second revision; Manuscript ID PGENETICS-D-25-00847

*Dear Editor,*

*We would like to sincerely thank you and the reviewers for the second favorable evaluation of our work and for the acceptance of our manuscript in PLOS Genetics.*

*We are pleased to submit the revised version of the manuscript, in which we have carefully addressed all comments raised by the reviewers.*

*Thank you again for your time and consideration*

*Yours sincerely*

*Richard Berthomé*

*Corresponding author*

**Journal Requirements**

If the reviewer comments include a recommendation to cite specific previously published works, please review and evaluate these publications to determine whether they are relevant and should be cited. There is no requirement to cite these works unless the editor has indicated otherwise.

1) Your manuscript's sections are not in the correct order.  Please amend to the following order: Abstract, Introduction, Results, Discussion, and Methods.

*We apologize for this formatting error. In the revised manuscript, we have corrected the placement of the Materials and Methods section and, consequently, adjusted the citation order of the references, as well as the numbering and citation order of all tables, figures, and Supplementary Information. These modifications are highlighted in the tracked-changes version of the manuscript.*

 2) Thank you for including the following in your data availability statement: 'The two reference genomes sequenced and annotated in this study have been deposited in the GenBank database under the accession numbers JASTWH000000000 (LA1246) and JASTWI000000000 (LA1670).' However, the accession number provided is currently inaccessible. Please verify and provide an active link to facilitate access to the data.

*Several co-authors have independently verified that the DOI links are functional. However, the accession numbers were still not resolving. These data were initially placed under embargo and we have requested that they be made publicly available. This process, which was expected to be rapid, is taking longer than anticipated. We are monitoring the situation regularly and will ensure that the records are accessible.* *In the worst-case scenario, if earlier release is not possible, the data will automatically become publicly available on 30 June 2026.*

**Responses to reviewer comments**

Reviewer #1:

1. Introduction, paragraph 3: This paragraph would benefit from revision for clarity and logical flow. At present, it includes many details about Arabidopsis defense mechanisms, which seem somewhat unnecessary in the context of this manuscript. More importantly, the main purpose of the paragraph is not clear. The reader may not understand whether the paragraph is intended to introduce QTLs/resistance genes or to justify the use of GWAS as an appropriate approach. Please consider rewriting this paragraph with a clearer focus.

*We thank the reviewer for the careful reading of our manuscript and for the constructive comments. This paragraph has been revised to improve clarity and strengthen the logical flow of the argument.*

2. Line 166: The text refers to “both temperatures,” but only 28°C appears to have been introduced in the paragraph. Please clarify or revise.

*The sentence has been revised to explicitly mention both temperatures (28°C and 32°C) and to clarify that the observed effect is associated with elevated temperature.*

3. Table S1 and line 192: In Table S1, the BIO6 column lists the lowest temperature as -28°C, whereas line 192 states -8°C. Please clarify which value is correct.

*We thank the reviewer for pointing out this error. The value reported in the previous version of line 192 was incorrect and has now been corrected. The revised sentence (lines 559-563) is consistent with the value reported in the supplementary material (-28.55°C), now presented in* ***S10 Table****.*

4. Line 320: Please clarify whether the correct number of accessions is 189 or 198.

*We thank the reviewer for this comment. We clarified in the section “Tomato panel composition and R. pseudosolanacearum strain” that the initially assembled panel consisted of 199 accessions (198 accessions + the ‘Hawaii’ control) that were phenotyped and that a subset of 189 accessions was selected, sequenced and used in all downstream analyses (lines 542-545).*

5. Line 365: Please specify what percentage of variation was considered.

*We thank the reviewer for this comment. The first three principal components included in the GWAS model explain 13.8% of the total variance when using the LA1670 reference genome (6.53% for PC1, 4.07% for PC2, and 2.68% for PC3) and 11.42% when using the LA1246 reference genome (5.53% for PC1, 3.52% for PC2, and 2.32% for PC3). This information has been added to the revised manuscript (lines 727-730).*

6. Line 413: Please remove the extra “S8.”

*The extra “S****8****” has been removed, in accordance with the renumbering of Supplementary* ***S8 Table*** *as* ***S11 Table*** *following the relocation of the Materials and Methods section (Line 778).*

7. Table 3: The temperature columns appear to be redundant, since panels A and B already correspond to specific temperatures. Please consider removing these columns or revising the table format.

*The columns have been removed from* ***Table 3****, and the legend has been corrected accordingly in the revised manuscript and in the file Belny et al_12062026_TabSTab.xlsx.*

8. Organization of figures and tables: In general, figures should be introduced in sequential order in the manuscript. However, the Results section begins by discussing Figure 3. Please consider reordering the figures and tables accordingly. In addition, Figure 5 is introduced for the first time in the Discussion; it should be presented and discussed in the Results section first.

*We thank the reviewer for this comment and fully agree with the concern raised. This issue was partly due to the previous placement of the Materials and Methods section in the manuscript, which affected the sequential citation of figures, tables, and supplementary materials in the results section. In the revised version, we have reorganized the manuscript to ensure that all figures, tables, and supplementary information are introduced in the appropriate sequential order.*

*In addition, the analyses and results designed to test the hypothesis of an environmental structuring of plant defense response-associated loci have been integrated into the “GWAS highlights temperature- and time-dependent biologically relevant candidate genes” section of the Results, where they are now associated with Figure 5, and the corresponding references to Figure 5 have been removed from the Discussion.*

9. Lines 614–626: Please add appropriate supporting citations, figures, or tables for the statements made in this section.

*We have addressed this comment in the revised manuscript by adding the appropriate supporting references.*

10. Figure 3, panels E and F: I am not sure whether panels E and F add substantial information to the figure. Since temperature and precipitation variation do not appear to cluster according to population structure, these panels seem somewhat redundant. Please consider moving them to the supplementary materials.

*We thank the reviewer for this comment. We consider that they provide a complementary, visually interpretation of the PCA axes. The projection of environmental variables onto the PCA space highlights broad geographic and climatic gradients that are consistent with both the spatial origin of the accessions and the known climatic characteristics of their collection areas, with an east–west gradient along Dim 1 and a north–south gradient along Dim 2. We use these panels as an illustrative element linking genetic variation with geographic and environmental context. For this reason, we would prefer to retain panels E and F in Figure 1.*

11. Figure legends: Please use a consistent format for introducing and describing figure panels across all figure legends.

*We thank the reviewer for this suggestion. The figure legends have been revised to ensure a consistent structure and formatting for introducing and describing figure panels. The corresponding changes are visible in the track-changes version of the revised manuscript. In addition, these modifications led us to adjust the legend of Fig. S7 to ensure consistency with the Results section.*

Reviewer #2:

It looks like the genbank numbers have not been updated. Similarly the links to the dois in the data statement are not active. This should be done before the paper can be accepted.

*We thank the reviewer for checking this point. As mentioned above, several co-authors have independently verified that the DOI links are functional. However, the GenBank accession numbers were still not resolving. These data were initially placed under embargo and we have requested that they be made publicly available. This process, which was expected to be rapid, is taking longer than anticipated.*

L450, percentages still dont add up to 100%. Does that mean the rest has missing data?
It may be that the altitude data are wrong in the database and for the conclusions of the manuscript it probably makes little difference, but the authors should mark this in their meta tables and not propagate error. Species ranges for pimpi en cerasiformae don't reach above 1500m.

*We thank the reviewer for this comment. This is indeed the case. Adding the two accessions for which available information indicates a collection altitude above 1500 m, which is unlikely (we have transformed these values as missing data (na) in our dataset to avoid propagating potentially erroneous information), the number of accessions for which no reliable information is available increases to 66, representing 34.9% of the panel. The adjusted percentages have been updated in the manuscript (lines 171–174).*
