# Supplementary material for: New biologically relevant resistance to bacterial wilt in heat-stressed tomato revealed by two-reference Genome Wide Association
Source: PLoS Genet. 2026 Aug 13;22(8):e1011820. doi: 10.1371/journal.pgen.1011820 (PMC13492982; doi:10.1371/journal.pgen.1011820)
Supplement: Peer review history

# Peer review: Decision Letter 0

**DOI:** 10.1371/journal.pgen.1011820.r001

**Authors:** Angela Hancock, Tatiana Giraud

9 Oct 2025

PGENETICS-D-25-00847

New resistance to bacterial wilt in heat-stressed tomato is revealed by two-reference Genome Wide Association

PLOS Genetics

Dear Dr. Berthomé,

Thank you for submitting your manuscript to PLOS Genetics. After careful consideration, we feel that it has merit but does not fully meet PLOS Genetics's publication criteria as it currently stands. Therefore, we invite you to submit a revised version of the manuscript that addresses the points raised during the review process.

Please submit your revised manuscript within 60 days Dec 08 2025 11:59PM. If you will need more time than this to complete your revisions, please reply to this message or contact the journal office at plosgenetics@plos.org. When you're ready to submit your revision, log on to https://www.editorialmanager.com/pgenetics/ and select the 'Submissions Needing Revision' folder to locate your manuscript file.

Please include the following items when submitting your revised manuscript:

* A rebuttal letter that responds to each point raised by the editor and reviewer(s). You should upload this letter as a separate file labeled 'Response to Reviewers'. This file does not need to include responses to any formatting updates and technical items listed in the 'Journal Requirements' section below.

* A marked-up copy of your manuscript that highlights changes made to the original version. You should upload this as a separate file labeled 'Revised Manuscript with Track Changes'.

* An unmarked version of your revised paper without tracked changes. You should upload this as a separate file labeled 'Manuscript'.

If you would like to make changes to your financial disclosure, competing interests statement, or data availability statement, please make these updates within the submission form at the time of resubmission. Guidelines for resubmitting your figure files are available below the reviewer comments at the end of this letter.

We look forward to receiving your revised manuscript.

Kind regards,

Tatiana Giraud

Academic Editor

PLOS Genetics

Angela Hancock

Section Editor

PLOS Genetics

Aimée Dudley

Editor-in-Chief

PLOS Genetics

Anne Goriely

Editor-in-Chief

PLOS Genetics

**Additional Editor Comments:**

We have now received three referee reports for your manuscript on the identification of loci for resistance to bacterial wilt in heat-stressed tomato by GWAS. All referees found the manuscript valuable and original; however, concerns were raised, in particular about the lack of clarity in the experimental design, the choice of accessions, statistical analyses, and data presentation, as well as lack of discussion of the study limitations. In addition, the referees suggest to tone down claims about gene functions, the existence of thermostable resistance and the utility of the two reference genomes, highlighting that the inference about so called "thermostable resistance" should actually be the opposite. They also recommend to discuss the findings in relationship with the accession origins, to better present the QTL results, with additional figures, and to perform a more comprehensive comparison of the two reference genomes.

I agree that the study would be a nice addition to PLoS Genetics but that the manuscript needs to be clarified and claims toned down. In addition, it would be better to begin the abstract and the introduction by general statements on the scientific topics rather than on specificities about the model organisms. Also, there are too many abbreviations, this renders the text harder to read without providing benefits. Please include page numbers. The analysis of population structure needs improvements and there are awkward statements on population genetics, see below some examples.

I would encourage resubmission if you are able to revise the manuscript along these lines. The referees also provide a list of excellent additional suggestions and questions, which should also be addressed.

More specific comments:

-L87: define PAMP

-P11-12: there are too many abbreviations and specific details, this renders the text harder to read

-L133-134; L166-167: unclear what you mean

-P14: unclear if these "wild relatives" all belong to a single species? a single population?

-L168: cut the sentence into two

-L375-7: the species involved should be clear from the introduction; the text mentions 5 species but only four are listed; why including different species, especially the two with a single accession?

-L386-390: unclear, two references are said to be needed because one species from different clusters, but what about the other species? The rationale for choosing different environments for a reference is unclear.

-Figure 1: unclear if these includes the five species and the correspondence with the clusters; the species delimitation could even be discussed given the lack of clear genetic subdivision; the figure and the text are unclear about how the clusters were delimited. Analyses such as STRCUTRE and Splitstree should be run. The delimitation into these 5 clusters is not convincing at all based on the PCAs.

-Figure 2: not sure this is needed as a main figure

-L431: it does change the separation of the five so called clusters

-L433: the term diversified is not correct here; give numbers of differences

-L436: you cannot say this without proper analysis and without convincingly showing genetic subdivision in the first place; the PCA lets rather think of a lack of clear genetic subdivision

-L439: the term "share" is not correct here, FST is a value for a pair

-Define all abbreviations in all figure legends

-L455-456: this is just the disease progressing, not susceptibility increasing

-L488: high, not important

-P33: a more comprehensive analysis of structural variants and better representation are needed

-L588: not exactly, the variation in the sample for the response to the disease was different at the two temperatures, but there may be a shared regulation that is not variable among accession

-L603: delete "interestingly"; based on what evidence was the previous annotation?

-L608: what does "particularly expressed » means? upregulated ?

-L632 : « assumption » is not the right term here, it is more a « hypothesis »

-L634 : unclear ; what is accentuated ?

-Do not refer to figures or Tables in the discussion, they should have explained well enough in the result section

-L637 : who is « they » ?

-L638, L674, L729: delete "interestingly";

-L639-643: this is a bit misleading, the results do not show well defined clusters in the response to disease, it looks more like a continuous trait homogeneous, saying it has lost diversity does not make sense, especially when using a single genome as a reference anyway

-L650: reduction in what?

-L614, L662: your question is unclear: do you look for resistance at high temperature, or at both temperature? This is different: clarify and discuss the results given the previse question

-L664: why is geographical distance important?

-L672: what do you mean by unique here?

-L671, 713, 736, 762, 771, 781: remove bold

-The discussion should end by broadening the context to other crops

**Journal Requirements:**

If the reviewer comments include a recommendation to cite specific previously published works, please review and evaluate these publications to determine whether they are relevant and should be cited. There is no requirement to cite these works unless the editor has indicated otherwise.

1) Please upload all main figures as separate Figure files in .tif or .eps format. For more information about how to convert and format your figure files please see our guidelines:

https://journals.plos.org/plosgenetics/s/figures

2) We have noticed that you have cited Table  2 in the manuscript file but there is no corresponding table in the manuscript.  Please amend your manuscript to include this table noting that tables should not be uploaded as individual files.

3) We have noticed that you have a list of Supporting Information legends in your manuscript. However, there are no corresponding files uploaded to the submission. Please upload them as separate files with the item type 'Supporting Information'.

4) Some material included in your submission may be copyrighted. According to PLOSu2019s copyright policy, authors who use figures or other material (e.g., graphics, clipart, maps) from another author or copyright holder must demonstrate or obtain permission to publish this material under the Creative Commons Attribution 4.0 International (CC BY 4.0) License used by PLOS journals. Please closely review the details of PLOSu2019s copyright requirements here: PLOS Licenses and Copyright. If you need to request permissions from a copyright holder, you may use PLOS's Copyright Content Permission form.

Please respond directly to this email and provide any known details concerning your material's license terms and permissions required for reuse, even if you have not yet obtained copyright permissions or are unsure of your material's copyright compatibility. Once you have responded and addressed all other outstanding technical requirements, you may resubmit your manuscript within Editorial Manager.

Potential Copyright Issues:

i) Figure 1. Please (a) provide a direct link to the base layer of the map (i.e., the country or region border shape) and ensure this is also included in the figure legend; and (b) provide a link to the terms of use / license information for the base layer image or shapefile. We cannot publish proprietary or copyrighted maps (e.g. Google Maps, Mapquest) and the terms of use for your map base layer must be compatible with our CC BY 4.0 license.

Note: if you created the map in a software program like R or ArcGIS, please locate and indicate the source of the basemap shapefile onto which data has been plotted.

If your map was obtained from a copyrighted source please amend the figure so that the base map used is from an openly available source. Alternatively, please provide explicit written permission from the copyright holder granting you the right to publish the material under our CC BY 4.0 license.

If you are unsure whether you can use a map or not, please do reach out and we will be able to help you. The following websites are good examples of where you can source open access or public domain maps:

* U.S. Geological Survey (USGS) - All maps are in the public domain. (http://www.usgs.gov)

* PlaniGlobe - All maps are published under a Creative Commons license so please cite u201cPlaniGlobe, http://www.planiglobe.com, CC BY 2.0u201d in the image credit after the caption. (http://www.planiglobe.com/?lang=enl)

* Natural Earth - All maps are public domain. (http://www.naturalearthdata.com/about/terms-of-use/).

5) Please amend your detailed Financial Disclosure statement. This is published with the article. It must therefore be completed in full sentences and contain the exact wording you wish to be published.

1) State the initials, alongside each funding source, of each author to receive each grant. For example: "This work was supported by the National Institutes of Health (####### to AM; ###### to CJ) and the National Science Foundation (###### to AM)."

2) State what role the funders took in the study. If the funders had no role in your study, please state: "The funders had no role in study design, data collection and analysis, decision to publish, or preparation of the manuscript."

3) If any authors received a salary from any of your funders, please state which authors and which funders..

If you did not receive any funding for this study, please simply state: u201cThe authors received no specific funding for this work.u201d

6) Kindly revise your competing statement in the online submission form to align with the journal's style guidelines: 'The authors declare that there are no competing interests.'

**Reviewers' comments:**

Reviewer's Responses to Questions

**Comments to the Authors:**

**Please note here if the review is uploaded as an attachment.**

Reviewer #1: Summary and significance:

This manuscript presents an ambitious GWAS of quantitative disease resistance (QDR) to Ralstonia solanacearumspecies complex in a panel of 189 wild tomato accessions. By phenotyping across two temperature regimes (28 °C vs 32 °C) and leveraging two de novo reference genomes (LA1246 and LA1670), the authors aim to identify candidate loci that may contribute to thermostable resistance under climate change. The dual-reference approach is innovative and has clear potential to complement pangenome efforts, and the daily scoring design provides temporal resolution rarely attempted in plant–pathogen GWAS. The work generates a valuable resource and important hypotheses.

That said, several aspects of the experimental design, statistical analysis, and data presentation require clarification or revision before the claims can be fully supported. My comments are divided into Major and Minor issues.

Major comments:

1. The authors emphasize thermostable resistance, yet no QTL were common to 28 °C and 32 °C. How do they justify the term “thermostable candidate loci” (see Abstract line ~57, Author summary) As field temperatures fluctuate below 28 °C and above 32 °C, it is unclear how results restricted to condition-specific QTL translate to breeding for stable resistance. Please clarify this interpretation.

2. Although the Results mention cases where SNPs could not be detected in LA1246, the Discussion does not explicitly address limitations. Please add a dedicated limitations paragraph.

3. In the Author summary, the authors state that some candidate loci are “currently undergoing functional validation.” If this is work planned by the authors, please move this point to a future directions statement in the Discussion.

4. The Introduction (lines ~152) states that optimal growth for tomato is 13–33 °C, yet 32 °C is used as the “heat stress” condition. Evidence is needed that the specific accessions studied have optimal growth below 32 °C. For example, a map of mean annual temperatures at accession origins would substantiate that 32 °C imposes stress relative to their natural ranges.

5. The phenotyping protocol (Materials & Methods) describes seed sterilization and transplanting but does not state clearly how many biological replicates per accession were used.

6. The inoculation method references Morel et al. (49) but does not specify whether root-dip, soil-drench, or stem injection was used. Please clarify. And the 0–4 disease index scale is not explained. A brief description of what each score corresponds to should be added.

7. The manuscript shows a Bonferroni line in Manhattan plots but does not explain the correction method.

8. Significant SNPs’ p-values are not reported in supplementary tables.

9. Given >3 million SNPs, Bonferroni is extremely strict (p ≈ 1.3 × 10⁻⁸). Consider also reporting FDR thresholds.

10. MAF was set to 0.032 (≈12 minor alleles in 189 diploids). This is low and risks unstable estimates. Please show the formula cited, explain why this cutoff was chosen.

11. Kinship estimation used filters A+B+C+D while GWAS used A+B+C+E. Please explain the rationale for using different SNP sets for kinship vs association.

12. Three PCs were included in GWAS; please report the variance explained by these PCs.

13. In the LA1246-based PCA, LA1246 and ‘Hawaii 7996’ cluster together. Is this due to introgression from S. pimpinellifolium into ‘Hawaii 7996’ or lack of sampling from S. lycopersicum? Please discuss.

14. Please provide a supplementary table with ancestry proportions of the five clusters, and consider comparing phenotypes across clusters to relate genetic diversity to resistance.

Minor comments:

o “Solanaceaous” → Solanaceae.

o Lines 86–93 (extended PTI vs ETI zig-zag model explanation) feel redundant and distract from the main story. Consider shortening.

o T3E is introduced at line 113 without prior definition. Please define “type III effectors (T3E)” when first mentioned (~line 103).

o Example: line 98 cites (3,2); line 108 cites (17,14). Please fix order and format.

o Figure 1 legend: clarify “A–C” should read “A & C” and “B–D” should read “B & D” to match panels. Also, each figure should indicate clearly which reference genome is used.

o Figure 3: the three response groups should be more clearly distinguished; adding representative plant images would strengthen the figure.

o Figure 5: Panel A y-axis should be labeled “local score.” Panel B legend refers to arrows that are not visible; please correct.

o Table 3: add a column with p-values for candidate SNPs/QTL.

o Results line 375: the panel included four species, not five (167 S. pimpinellifolium, 20 S. lycopersicum var. cerasiforme, one S. cheesmaniae, one S. lycopersicum cultivar). Please correct.

o Please state whether any non-inoculated wild accessions were grown at both temperatures to confirm symptoms were disease-related and not physiological stress.

o “GWA” (line 483) should be written consistently as “GWAS.”

Overall assessment:

This is an important and timely study. The innovative use of two panel-derived reference genomes to improve QTL detection is a real strength. However, clarification of experimental replication, inoculation methods, statistical thresholds, and explicit acknowledgment of limitations will be required to ensure reproducibility and to temper the claims about thermostable resistance. With these revisions, the manuscript will be a valuable contribution to the field.

Reviewer #2: This study presents a high-quality genomic and phenotypic analysis of 189 wild tomato accessions, combining two newly sequenced reference genomes with a temperature-controlled GWAS approach to investigate resistance to R. solanacearum. The work reveals substantial natural variation in bacterial wilt responses, identifies temperature- and time-dependent QTLs, and highlights candidate genes with potential roles in immunity, calcium signaling, and receptor-mediated defense. Whreas true validation of the results is lacking, overall, the study provides a valuable genomic resource and a well-characterized panel for uncovering thermostable resistance mechanisms in tomato.

I only have some minor comments:

-Not sure why the data set was reduced from 199 to 189

-Percentages of the altitude of origin don’t add up.

-It is extremely unlikely to find Pimpinellifolium or Cerasiformae above 3000 m. Please check your records again.

-You first report 21 candidates, later only 19 homologs?

-Can you authors explain a bit more why it is not weird that there was no overlap between QTL? Only 3 degrees difference?

-Did the authors test whether the plants were stressed beforeand like by measuring Fv/Fm or other measures of respiratory fitness?

-Whereas the study is useful, claiming using two reference for different GWAS is not the same as offering alternatives for Pangenomes, the relative sections should be tuned down

-The functional claims of the GWAS candidates are solely based on Arabidopsis annotations. This should be properly placed in context without overstatement

- The discussion could be tuned down. There were no actually validated candidate genes. I also don’t think that is needed for this study, but until real validation happens… I also think the time and temperature dependent results should be discussed a bit more in light of possible false positives or why the authors think all these loci are real.

Reviewer #3: The review is in attachment

**********

**Have all data underlying the figures and results presented in the manuscript been provided?**

Large-scale datasets should be made available via a public repository as described in the *PLOS Genetics*  data availability policy, and numerical data that underlies graphs or summary statistics should be provided in spreadsheet form as supporting information.

Reviewer #1: **No:** The SNPs P-value from GWAS study is missing

Reviewer #2: None

Reviewer #3: Yes

**********

PLOS authors have the option to publish the peer review history of their article (what does this mean?). If published, this will include your full peer review and any attached files.

If you choose “no”, your identity will remain anonymous but your review may still be made public.

**Do you want your identity to be public for this peer review?**  For information about this choice, including consent withdrawal, please see our Privacy Policy.

Reviewer #1: No

Reviewer #2: No

Reviewer #3: No

[NOTE: If reviewer comments were submitted as an attachment file, they will be attached to this email and accessible via the submission site. Please log into your account, locate the manuscript record, and check for the action link "View Attachments". If this link does not appear, there are no attachment files.]

**Figure resubmission:**

While revising your submission, we strongly recommend that you use PLOS’s NAAS tool (https://ngplosjournals.pagemajik.ai/artanalysis) to test your figure files. NAAS can convert your figure files to the TIFF file type and meet basic requirements (such as print size, resolution), or provide you with a report on issues that do not meet our requirements and that NAAS cannot fix.

After uploading your figures to PLOS’s NAAS tool - https://ngplosjournals.pagemajik.ai/artanalysis, NAAS will process the files provided and display the results in the "Uploaded Files" section of the page as the processing is complete. If the uploaded figures meet our requirements (or NAAS is able to fix the files to meet our requirements), the figure will be marked as "fixed" above. If NAAS is unable to fix the files, a red "failed" label will appear above. When NAAS has confirmed that the figure files meet our requirements, please download the file via the download option, and include these NAAS processed figure files when submitting your revised manuscript.

**Reproducibility:**

To enhance the reproducibility of your results, we recommend that authors of applicable studies deposit laboratory protocols in protocols.io, where a protocol can be assigned its own identifier (DOI) such that it can be cited independently in the future. Additionally, PLOS ONE offers an option to publish peer-reviewed clinical study protocols. Read more information on sharing protocols at https://plos.org/protocols?utm_medium=editorial-email&utm_source=authorletters&utm_campaign=protocols

Attachment Submitted filename: PGENETICS-D-25-00847_review.docx

Attachment Submitted filename: PGENETICS-D-25-00847_reviewer.pdf

# Author comment: Author response to Decision Letter 1

**DOI:** 10.1371/journal.pgen.1011820.r002

15 Apr 2026

Attachment Submitted filename: Responses to Reviewers.docx

# Peer review: Decision Letter 1

**DOI:** 10.1371/journal.pgen.1011820.r003

**Authors:** Angela Hancock, Tatiana Giraud

13 May 2026

PGENETICS-D-25-00847R1

New biologically relevant resistance to bacterial wilt in heat-stressed tomato revealed by two-reference Genome Wide Association

PLOS Genetics

Dear Dr. Berthomé,

Thank you for submitting your manuscript to PLOS Genetics. After careful consideration, we feel that it has merit but does not fully meet PLOS Genetics's publication criteria as it currently stands. Therefore, we invite you to submit a revised version of the manuscript that addresses the points raised during the review process.

Please submit your revised manuscript within by Jun 12 2026 11:59PM. If you will need more time than this to complete your revisions, please reply to this message or contact the journal office at plosgenetics@plos.org. When you're ready to submit your revision, log on to https://www.editorialmanager.com/pgenetics/ and select the 'Submissions Needing Revision' folder to locate your manuscript file.

Please include the following items when submitting your revised manuscript:

* A letter that responds to each point raised by the editor and reviewer(s). You should upload this letter as a separate file labeled 'Response to Reviewers'. This file does not need to include responses to formatting updates and technical items listed in the 'Journal Requirements' section below.

* A marked-up copy of your manuscript that highlights changes made to the original version. You should upload this as a separate file labeled 'Revised Manuscript with Track Changes'.

* An unmarked version of your revised paper without tracked changes. You should upload this as a separate file labeled 'Manuscript'.

If you would like to make changes to your financial disclosure, competing interests statement, or data availability statement, please make these updates within the submission form at the time of resubmission. Guidelines for resubmitting your figure files are available below the reviewer comments at the end of this letter.

As the corresponding author, your ORCID iD is verified in the submission system and will appear in the published article. PLOS supports the use of ORCID, and we encourage all coauthors to register for an ORCID iD and use it as well. Please encourage your coauthors to verify their ORCID iD within the submission system before final acceptance, as unverified ORCID iDs will not appear in the published article. Only the individual author can complete the verification step; PLOS staff cannot verify ORCID iDs on behalf of authors.

We look forward to receiving your revised manuscript.

Kind regards,

Tatiana Giraud

Academic Editor

PLOS Genetics

Aimée Dudley

Editor-in-Chief

PLOS Genetics

Anne Goriely

Editor-in-Chief

PLOS Genetics

**Additional Editor Comments:**

I was pleased to see that this manuscript has been carefully revised. The referees also found the revisions overall satisfactory, but they had additional suggestions left. Other than that, I congratulate the authors for this nice piece of work that will be a great contribution to the journal.

**Journal Requirements:**

If the reviewer comments include a recommendation to cite specific previously published works, please review and evaluate these publications to determine whether they are relevant and should be cited. There is no requirement to cite these works unless the editor has indicated otherwise.

1) Your manuscript's sections are not in the correct order.  Please amend to the following order: Abstract, Introduction, Results, Discussion, and Methods.

2) ) Thank you for including the following in your data availability statement: 'The two reference genomes sequenced and annotated in this study have been deposited in the GenBank database under the accession numbers JASTWH000000000 (LA1246) and JASTWI000000000 (LA1670).' However, the accession number provided is currently inaccessible. Please verify and provide an active link to facilitate access to the data.

**Reviewers' comments:**

Reviewer's Responses to Questions

**Comments to the Authors:**

**Please note here if the review is uploaded as an attachment.**

Reviewer #1: The manuscript presents valuable results and makes a meaningful contribution to our understanding of plant–pathogen interactions in tomato, an important model crop. The authors have also responded to the reviewers’ comments fairly.

My recommendation is acceptance with minor revisions. My remaining comments are listed below:

1. Introduction, paragraph 3: This paragraph would benefit from revision for clarity and logical flow. At present, it includes many details about Arabidopsis defense mechanisms, which seem somewhat unnecessary in the context of this manuscript. More importantly, the main purpose of the paragraph is not clear. The reader may not understand whether the paragraph is intended to introduce QTLs/resistance genes or to justify the use of GWAS as an appropriate approach. Please consider rewriting this paragraph with a clearer focus.

2. Line 166: The text refers to “both temperatures,” but only 28°C appears to have been introduced in the paragraph. Please clarify or revise.

3. Table S1 and line 192: In Table S1, the BIO6 column lists the lowest temperature as -28°C, whereas line 192 states -8°C. Please clarify which value is correct.

4. Line 320: Please clarify whether the correct number of accessions is 189 or 198.

5. Line 365: Please specify what percentage of variation was considered.

6. Line 413: Please remove the extra “S8.”

7. Table 3: The temperature columns appear to be redundant, since panels A and B already correspond to specific temperatures. Please consider removing these columns or revising the table format.

8. Organization of figures and tables: In general, figures should be introduced in sequential order in the manuscript. However, the Results section begins by discussing Figure 3. Please consider reordering the figures and tables accordingly. In addition, Figure 5 is introduced for the first time in the Discussion; it should be presented and discussed in the Results section first.

9. Lines 614–626: Please add appropriate supporting citations, figures, or tables for the statements made in this section.

10. Figure 3, panels E and F: I am not sure whether panels E and F add substantial information to the figure. Since temperature and precipitation variation do not appear to cluster according to population structure, these panels seem somewhat redundant. Please consider moving them to the supplementary materials.

11. Figure legends: Please use a consistent format for introducing and describing figure panels across all figure legends.

Overall, the manuscript is valuable and suitable for publication after these minor revisions are addressed.

Reviewer #2: The manuscript is much improved. The new discussion nicely incorporates comments from all reveiwers and editors.

It looks like the genbank numbers have not been updated. Similarly the links to the dois in the data statement are not active.

This should be done before the paper can be accepted.

l450, percentages still dont add up to 100%. Does that mean the rest has missing data?

It may be that the altitude data are wrong in the database and for the conclusions of the manuscript it probably makes little difference, but the authors should mark this in their meta tables and not propagate error. Species ranges for pimpi en cerasiformae don't reach above 1500m.

Reviewer #3: Thank you to the authors for their hard work on this update. All requested modifications have been successfully integrated, and I am pleased to accept the document in its current form.

**********

**Have all data underlying the figures and results presented in the manuscript been provided?**

Large-scale datasets should be made available via a public repository as described in the *PLOS Genetics*  data availability policy, and numerical data that underlies graphs or summary statistics should be provided in spreadsheet form as supporting information.

Reviewer #1: Yes

Reviewer #2: **No:**

Reviewer #3: Yes

**********

PLOS authors have the option to publish the peer review history of their article (what does this mean?). If published, this will include your full peer review and any attached files.

If you choose “no”, your identity will remain anonymous but your review may still be made public.

**Do you want your identity to be public for this peer review?**  For information about this choice, including consent withdrawal, please see our Privacy Policy.

Reviewer #1: No

Reviewer #2: No

Reviewer #3: No

[NOTE: If reviewer comments were submitted as an attachment file, they will be attached to this email and accessible via the submission site. Please log into your account, locate the manuscript record, and check for the action link "View Attachments". If this link does not appear, there are no attachment files.]

**Figure resubmission:**

While revising your submission, we strongly recommend that you use PLOS’s NAAS tool (https://ngplosjournals.pagemajik.ai/artanalysis) to test your figure files. NAAS can convert your figure files to the TIFF file type and meet basic requirements (such as print size, resolution), or provide you with a report on issues that do not meet our requirements and that NAAS cannot fix.

After uploading your figures to PLOS’s NAAS tool - https://ngplosjournals.pagemajik.ai/artanalysis, NAAS will process the files provided and display the results in the "Uploaded Files" section of the page as the processing is complete. If the uploaded figures meet our requirements (or NAAS is able to fix the files to meet our requirements), the figure will be marked as "fixed" above. If NAAS is unable to fix the files, a red "failed" label will appear above. When NAAS has confirmed that the figure files meet our requirements, please download the file via the download option, and include these NAAS processed figure files when submitting your revised manuscript.

**Reproducibility:**

To enhance the reproducibility of your results, we recommend that authors deposit laboratory protocols in protocols.io, where a protocol can be assigned its own identifier (DOI) such that it can be cited independently in the future. Additionally, PLOS ONE offers an option to publish peer-reviewed clinical study protocols. Read more information on sharing protocols at https://plos.org/protocols?utm_medium=editorial-email&utm_source=authorletters&utm_campaign=protocols

# Author comment: Author response to Decision Letter 2

**DOI:** 10.1371/journal.pgen.1011820.r004

12 Jun 2026

Attachment Submitted filename: Responses to Reviewers_second revision.docx

# Editor report: Decision Letter 2

**DOI:** 10.1371/journal.pgen.1011820.r005

**Authors:** Angela Hancock, Tatiana Giraud

17 Jun 2026

Dear Dr Berthomé,

We are pleased to inform you that your manuscript entitled "New biologically relevant resistance to bacterial wilt in heat-stressed tomato revealed by two-reference Genome Wide Association " has been editorially accepted for publication in PLOS Genetics. Congratulations!

Before your submission can be formally accepted and sent to production you will need to complete our formatting changes, which you will receive in a follow up email. Please be aware that it may take several days for you to receive this email; during this time no action is required by you. Please note: the accept date on your published article will reflect the date of this provisional acceptance, but your manuscript will not be scheduled for publication until the required changes have been made.

Once your paper is formally accepted, an uncorrected proof of your manuscript will be published online ahead of the final version, unless you’ve already opted out via the online submission form. If, for any reason, you do not want an earlier version of your manuscript published online or are unsure if you have already indicated as such, please let the journal staff know immediately at plosgenetics@plos.org.

In the meantime, please log into Editorial Manager at https://www.editorialmanager.com/pgenetics/, click the "Update My Information" link at the top of the page, and update your user information to ensure an efficient production and billing process. Note that PLOS requires an ORCID iD for all corresponding authors. Therefore, please ensure that you have an ORCID iD and that it is validated in Editorial Manager. To do this, go to ‘Update my Information’ (in the upper left-hand corner of the main menu), and click on the Fetch/Validate link next to the ORCID field.  This will take you to the ORCID site and allow you to create a new iD or authenticate a pre-existing iD in Editorial Manager.

If you have a press-related query, or would like to know about making your underlying data available (as you will be aware, this is required for publication), please see the end of this email. If your institution or institutions have a press office, please notify them about your upcoming article at this point, to enable them to help maximise its impact. Inform journal staff as soon as possible if you are preparing a press release for your article and need a publication date.

Thank you again for supporting open-access publishing; we are looking forward to publishing your work in PLOS Genetics!

Yours sincerely,

Tatiana Giraud

Academic Editor

PLOS Genetics

Angela Hancock

Section Editor

PLOS Genetics

Aimée Dudley

Editor-in-Chief

PLOS Genetics

Anne Goriely

Editor-in-Chief

PLOS Genetics

www.plosgenetics.org

BlueSky: @plos.bsky.social

----------------------------------------------------

Comments from the reviewers (if applicable):

----------------------------------------------------

**Data Deposition**

If you have submitted a Research Article or Front Matter that has associated data that are not suitable for deposition in a subject-specific public repository (such as GenBank or ArrayExpress), one way to make that data available is to deposit it in the Dryad Digital Repository. As you may recall, we ask all authors to agree to make data available; this is one way to achieve that. A full list of recommended repositories can be found on our website.

The following link will take you to the Dryad record for your article, so you won't have to re‐enter its bibliographic information, and can upload your files directly:

http://datadryad.org/submit?journalID=pgenetics&manu=PGENETICS-D-25-00847R2

More information about depositing data in Dryad is available at http://www.datadryad.org/depositing. If you experience any difficulties in submitting your data, please contact help@datadryad.org for support.

Additionally, please be aware that our data availability policy requires that all numerical data underlying display items are included with the submission, and you will need to provide this before we can formally accept your manuscript, if not already present.

----------------------------------------------------

**Press Queries**

If you or your institution will be preparing press materials for this manuscript, or if you need to know your paper's publication date for media purposes, please inform the journal staff as soon as possible so that your submission can be scheduled accordingly. Your manuscript will remain under a strict press embargo until the publication date and time. This means an early version of your manuscript will not be published ahead of your final version. PLOS Genetics may also choose to issue a press release for your article. If there's anything the journal should know or you'd like more information, please get in touch via plosgenetics@plos.org.

# Editor report: Acceptance letter

**DOI:** 10.1371/journal.pgen.1011820.r006

**Authors:** Angela Hancock, Tatiana Giraud

PGENETICS-D-25-00847R2

New biologically relevant resistance to bacterial wilt in heat-stressed tomato revealed by two-reference Genome Wide Association

Dear Dr Berthomé,

We are pleased to inform you that your manuscript entitled "

New biologically relevant resistance to bacterial wilt in heat-stressed tomato revealed by two-reference Genome Wide Association " has been formally accepted for publication in PLOS Genetics! Your manuscript is now with our production department and you will be notified of the publication date in due course.

The corresponding author will soon be receiving a typeset proof for review, to ensure errors have not been introduced during production. Please review the PDF proof of your manuscript carefully, as this is the last chance to correct any errors. Please note that major changes, or those which affect the scientific understanding of the work, will likely cause delays to the publication date of your manuscript.

Soon after your final files are uploaded, unless you have opted out or your manuscript is a front-matter piece, the early version of your manuscript will be published online. The date of the early version will be your article's publication date. The final article will be published to the same URL, and all versions of the paper will be accessible to readers.

For Research Articles, you will receive an invoice from PLOS for your publication fee after your manuscript has reached the completed accept phase. If you receive an email requesting payment before acceptance or for any other service, this may be a phishing scheme. Learn how to identify phishing emails and protect your accounts at https://explore.plos.org/phishing.

Thank you again for supporting PLOS Genetics and open-access publishing. We are looking forward to publishing your work!

With kind regards,

Sharmila Kamatchi

PLOS Genetics

On behalf of:

The PLOS Genetics Team

Carlyle House, Carlyle Road, Cambridge CB4 3DN | United Kingdom

plosgenetics@plos.org | +44 (0) 1223-442823

plosgenetics.org | Twitter: @PLOSGenetics
